# Structure, kinetic characterization and subcellular localization of the two ribulose 5-phosphate epimerase isoenzymes from *Trypanosoma cruzi*

**DOI:** 10.1371/journal.pone.0172405

**Published:** 2017-02-16

**Authors:** Soledad Natalia Gonzalez, Wanda Mariela Valsecchi, Dante Maugeri, José María Delfino, Juan José Cazzulo

**Affiliations:** 1 Instituto de Investigaciones Biotecnológicas Dr. Rodolfo A. Ugalde-Instituto Tecnológico de Chascomús Dr. Raúl Alfonsín (IIB-INTECH), Universidad Nacional de San Martín (UNSAM)-Consejo Nacional de Investigaciones Científicas y Técnicas (CONICET), Campus Miguelete, Buenos Aires, Argentina; 2 Instituto de Química y Fisicoquímica Biológicas, Universidad de Buenos Aires, Junín, Buenos Aires, Argentina; Instituto Nacional de Cardiologia, MEXICO

## Abstract

The enzyme of the pentose phosphate pathway (PPP) ribulose-5-phosphate-epimerase (RPE) is encoded by two genes present in the genome of *Trypanosoma cruzi* CL Brener clone: *TcRPE1* and *TcRPE2*. Despite high sequence similarity at the amino acid residue level, the recombinant isoenzymes show a strikingly different kinetics. Whereas TcRPE2 follows a typical michaelian behavior, TcRPE1 shows a complex kinetic pattern, displaying a biphasic curve, suggesting the coexistence of -at least- two kinetically different molecular forms. Regarding the subcellular localization in epimastigotes, whereas TcRPE1 is a cytosolic enzyme, TcRPE2 is localized in glycosomes. To our knowledge, TcRPE2 is the first PPP isoenzyme that is exclusively localized in glycosomes. Over-expression of TcRPE1, but not of TcRPE2, significantly reduces the parasite doubling time *in vitro*, as compared with wild type epimastigotes. Both TcRPEs represent single domain proteins exhibiting the classical α/β TIM-barrel fold, as expected for enzymes with this activity. With regard to the architecture of the active site, all the important amino acid residues for catalysis -with the exception of M58- are also present in both TcRPEs models. The superimposition of the binding pocket of both isoenzyme models shows that they adopt essentially identical positions in the active site with a residue specific RMSD < 2Å, with the sole exception of S12, which displays a large deviation (residue specific RMSD: 11.07 Å). Studies on the quaternary arrangement of these isoenzymes reveal that both are present in a mixture of various oligomeric species made up of an even number of molecules, probably pointing to the dimer as their minimal functional unit. This multiplicity of oligomeric species has not been reported for any of the other RPEs studied so far and it might bear implications for the regulation of TcRPEs activity, although further investigation will be necessary to unravel the physiological significance of these structural findings.

## Introduction

The pentose phosphate pathway (PPP) is a metabolic route that starts with glucose-6-phosphate, the first glycolytic intermediate. It consists of two branches, the oxidative branch leading from glucose 6-phosphate to ribulose-5-phosphate (Ru5P), with the reduction of two molecules of NADP^+^, and the non-oxidative or sugar interconversion branch, which ultimately, when functioning as a cycle, leads back to glycolytic intermediates. The PPP usually plays two major roles, namely the reduction of NADP^+^ to NADPH, a coenzyme necessary for biosynthetic reactions and for the protection of cells against oxidative stress imposed by reactive oxygen species (ROS), as well as the production of ribose-5-phosphate to be used in nucleic acid synthesis [1].

Ribulose-5-phosphate epimerase (RPE), which catalyses the reversible conversion of Ru5P to xylulose-5-phosphate (Xu5P), is an enzyme of the non-oxidative branch of the PPP. RPEs are broadly distributed in the three kingdoms of life, including most eukaryotic organisms, fungi and bacteria [2–7]. Little is known about RPEs kinetic properties, but RPE is generally considered a metalloenzyme in several of the studied organisms and has been shown to use divalent cations as cofactors or activators [8–11]. As a metalloenzyme, RPE in *E*.*coli* is thought to be one of the principal targets of oxidative stress generated by H_2_O_2_ [12,13]. RPEs belong to the ribulose phosphate binding superfamily, sharing a common (*α*/β) 8-strand barrel fold and a conserved phosphate binding motif located at the end of the seventh and eighth β-strands in most of the members of this family [11]. As for its quaternary structure, RPEs are oligomers made up of an even number of subunits in most of the organisms studied [3,7–11,14–17].

*Trypanosoma cruzi*, the parasitic protozoan that causes the American Trypanosomiasis (Chagas disease) maintains a functional PPP [18]. The seven enzymes of the pathway are present in the four major developmental stages of the parasite: the intracellular amastigote and the bloodstream trypomastigote in the mammalian host, and the epimastigote and metacyclic trypomastigote, in the insect vector [19]. Subcellular localization experiments suggested that the PPP enzymes display a predominant cytosolic component, although in all cases they also seem to show a minor particulate localization [19].

In several protozoan parasites, enzymes belonging to the oxidative branch of the PPP have been extensively studied [20–27], but information is scarce on most of the enzymes of the non-oxidative branch, with the exception of ribose-5-phosphate isomerase from *T*. *cruzi* [28,29], *Leishmania donovani* [30–33] and *T*. *brucei* [34]; and transketolase from *Leishmania mexicana* and *T*.*brucei* [35–37]. In the present work, we report research on the kinetic properties of the recombinant TcRPE isoenzymes from *T*. *cruzi*, as well as a molecular modeling study that highlights important functional features of their tertiary structure, and evidence on their quaternary arrangement obtained by light scattering. Through several different techniques, we were also able to analyze the subcellular localization of the native and recombinant (over-expressed) TcRPEs in the epimastigote developmental stage.

## Materials and methods

### Parasites growth

Epimastigotes of the *T*. *cruzi* CL Brener clone were grown in axenic medium, harvested and washed as previously described [38]. Cell-free extracts from epimastigotes were obtained by three cycles of freezing and thawing, and then suspension was performed in buffer 1 (50 mM Tris–HCl buffer, pH 7.6, containing 1 mM EDTA and 0.25 M sucrose) in the presence of 0.5 mM tosyl-lysyl-chloromethyl ketone (TLCK). Then the cell-free extracts were treated by sonication in a Branson 450 Sonifier, by three continuous pulses (30-s each) at 60% of maximal power. The suspensions were centrifuged at 20,000 x g for 10 min, and the supernatants were used for the determination of enzymatic activity and preparation of Western blots.

### Cloning of TcRPE1 and TcRPE2

The *TcRPE1* ORF (Accession code: EU075265.1) of 735 bp and *TcRPE2* ORF (Accession code: EU075266.1) of 744 bp, were amplified by PCR from genomic DNA from *T*. *cruzi* CL Brener clone epimastigotes. Primers were designed according to the sequence data obtained from GenBank^™^ database. NdeI and XhoI sites were included to facilitate the directional cloning into the expression vector. The sequences of the primers were as follows: sense primer **TcRPE1FW:** 5'-CAT**ATG**TTTGATCACCAGGACCG-3' and sense primer **TcRPE2FW:**5'-CAT**ATG**CGCAGCTTTGATCATCAG-3' (start bold, NdeI underlined); antisense primer **TcRPE1RV:** 5'-CTCGAGTTTTGGGGTGGAGACTTCTTAC-3' and antisense primer **TcRPE2RV:** CTCGAGAAGGTGGGATTTCAAGTGGTGG-3' (XhoI underlined). PCR conditions were as follows: initial denaturation (300 s at 95°C), 30 cycles of denaturation (30 s at 95°C), annealing (30 s at 58°C), elongation (60 s at 68°C) and a final extension step (90 s at 68°C). The PCR products were isolated from a 1% agarose gel, purified by the QIAquick PCR purification kit protocol (Qiagen), and cloned into a pGEM^®^-T Easy vector (Promega). Sequencing of the products was performed using the sequencing service provided by MACROGEN (South Korea).

### Construction of vector pET-22b(+)-TcRPE1 / TcRPE2, expression, and purification of recombinant TcRPEs

The *TcRPE1* and *TcRPE2* genes were excised as NdeI/XhoI fragments from the pGEM^®^-T Easy vector, gel purified and subcloned into the NdeI and XhoI sites of the pET-22b(+) expression vector (Novagen). The resulting constructs presenting a poly-His tag at the C-terminus were transferred to *E*. *coli* BL21 codon Plus (DE3) cells. Transformations with pET-22b(+)-*TcRPE1* and pET-22b(+)-*TcRPE2* were performed according to procedures described in the instruction manual. For expression, a single colony was grown overnight at 37°C in LB medium containing 50 μg ml^−1^ kanamycin and 10 μg ml^−1^ chloramphenicol. Then, cells were diluted 1:25 in LB medium [39] containing antibiotics as described above, and grown at 37°C. When an optical density of 0.6 at 600 nm was reached, isopropyl-β-D-thiogalactopyranoside (IPTG) was added to a final concentration of 0.5 mM to induce protein expression. Then, growth was continued at 18°C overnight with shaking at 250 rev min^−1^. Cells were harvested by centrifugation at 3,000 x g for 10 min at 4°C and resuspended in lysis buffer containing: 50 mM Tris-HCl, pH 7.5, 500 mM NaCl, 0.1% Triton X-100, 2 mM PMSF, and lysozyme at a final concentration of 1 mg ml^−1^. DNA was digested by treatment with DNase I (0.1 mg ml^−1^ final concentration). The cell-free extract was obtained by centrifugation at 20,000 x g for 25 min at 4°C.

The recombinant enzymes were purified in one step using Ni^2+^ resin (GE Healthcare) pre-equilibrated in 50 mM Tris-HCl (pH 7.6) containing 500 mM NaCl. The column was washed sequentially with 50 column volumes (CV) of the equilibration buffer and 50 CV of the same buffer added with 50 mM imidazole. TcRPE1 and TcRPE2 were eluted with 2 CV of the equilibrium buffer added with 300 mM imidazole and 500 μl fractions were collected. All purification procedures were performed at 4°C and the elution profile was monitored by enzymatic activity. Purity of the recombinant TcRPEs was analyzed by SDS-PAGE followed by Coomassie Blue staining. The eluted fractions were then pooled and desalted by following the PD-10 desalting column protocol (GE Healthcare), using equilibrium buffer as desalting buffer.

### Cloning, expression and purification of T.cruzi transketolase

Since production of the commercial transketolase (TKT) that we used in the beginning as a coupled enzyme for the RPE assay was discontinued, we decided to produce the enzyme from *T*.*cruzi*. The *TcTKT* ORF (Accession code: EU077555.1) of 2019 bp, was amplified by PCR from genomic DNA from *T*. *cruzi* CL Brener clone epimastigotes. Primers were designed according to the sequence data obtained from GenBank^™^ database. NheI and XhoI sites were included to facilitate the directional cloning into the expression vector. The sequences of the primers were as follows: sense primer **TcTKTFW**: 5'-CGTACG**ATG**AATAACAGTAAA-3' (start bold, NheI underlined) and **TcTKTRV**: 5'-CTCGAGTCACAGATGCACACG-3' (XhoI underlined). PCR conditions were as follows: initial denaturation (300 s at 95°C), 30 cycles of denaturation (30 s at 95°C), annealing (60 s at 58°C), elongation (150 s at 68°C) and a final extension step (150 s at 68°C). The PCR products were isolated from a 1% agarose gel, purified by the QIAquick PCR purification kit protocol (Qiagen), and cloned into a pGEM^®^-T Easy vector (Promega). Sequencing of the products was performed by using TcTKTFW, TcTKTRV and the internal primers: **TcTKTFW5**: 5'-ACGCGCTTGGTGCTG-3', **TcTKTRV3**: 5'-CTCATTGGTGGATCTTCGGA-3' and **TcTKTRV4**: 5'- GCTACGCGCTTGGTGCTG-3' by the automated sequencing service provided by MACROGEN (South Korea). The *TcTKT* gene was excised as NheI/XhoI fragments from the pGEM^®^-T Easy vector, gel purified and subcloned into the NheI and XhoI sites of the pET-28a(+) expression vector (Novagen). Then, we followed the same protocol described to express and purify the recombinant TcRPE1 and 2. TcTKT active enzyme was purified to homogeneity and stored in glycerol 30% at -80°C for its use as a coupled enzyme for the determination of RPE activity.

### Fractionation by differential centrifugation

Epimastigotes of the CL Brener clone were disrupted in a mortar using silicon carbide, in a ratio of 2 g per g of cells, wet weight. The cell paste was suspended in buffer 1 (see above) and the suspension was centrifuged for 3 min at 100 x g to remove the abrasive powder. The total homogenate (H) was submitted to fractionation by differential centrifugation [19] and three fractions were obtained, namely large granules (LG), small granules (SG) and the final supernatant (S). The resultant SG fraction was used to perform a sucrose linear density gradient centrifugation (see below).

### Sucrose linear density gradient centrifugation

Linear and continuous gradients from 0.5 to 2.0 M sucrose in 50 mM Tris-HCl pH 7.6, 1mM EDTA, were prepared in polyallomer tubes of 16 mm x 67 mm (10.75 ml capacity) by the successive addition of layers of sucrose solutions of decreasing density, and the system was left to equilibrate overnight. Then, the SG fraction (0.25 ml) -obtained by fractionation by differential centrifugation—was softly homogenized to eliminate any association among particles, and loaded over the gradient surface. The volume of the tube was completed with 50 mM Tris-HCl, pH 7.6, 1mM EDTA, and then the tube was heat sealed. Immediately, the tube was placed in a NVT 65 rotor and centrifuged at 300,000 x g for 3 h. In the end, 23 fractions (0.5 ml each) were collected by suction, using an automatic collector. Fraction density was determined with an Abbe refractometer. All the operations were performed between 0 and 4°C. The activities of RPE and marker enzymes were determined in reaction mixtures containing 0.2% Triton X-100 and were represented in frequency histograms, as the total activity of a given fraction relative to the sum of total activities of all the fractions, as a function of the fraction number ordered by increasing density.

### Enzyme assays

All enzyme assays were performed at 30°C in a Beckman DU600 spectrophotometer. RPE activity was assayed by a coupled enzyme method, as previously described [35] except that TcRPE1 and TcRPE2 were assayed at pH 7.25 and 7.5, respectively. A concentration of 0.02–2 mM or 0.1–10 mM D-ribulose-5-phosphate was assayed for recombinant TcRPE1 or TcRPE2, respectively. The TcTKT used as coupled enzyme, was assayed as described in [35]. The other coupled enzymes were purchased from Sigma Aldrich (St. Louis, MO). The values of the kinetic parameters K_M_ and k_cat_ for TcRPE2 were obtained after non-linear regression of velocities in the substrate curves by the Gauss-Newton method, using the Solver application from Microsoft Office Excel. The data of four independent experiments performed with different batches of TcRPE2, were fitted using the Michaelis-Menten equation. In a similar fashion, the data of four independent experiments performed with different batches of TcRPE1, were plotted to determine the complex kinetic pattern shown by this enzyme. Hence, we decided not to apply any mathematical treatment to these data. The glycosomal marker hexokinase was assayed as described in [35]. The mitochondrial marker isocitrate dehydrogenase was assayed in a reaction mixture containing 2 mM MnCl_2_, 0.5 mM NADP and 2.5 mM isocitrate in 25 mM triethanolamine-HCl buffer (pH 7.5). The reaction was monitored by reading the increase in the absorbance at 340 nm. The Golgi apparatus marker acid phosphatase was assayed at pH 5.4 in a reaction mixture containing 150 mM citrate, 200 mM pentanediol, 12.5 mM α-naphthyl phosphate and 75 mM Fast Red TR. The reaction was monitored by reading the increase in the absorbance at 405 nm. The protein concentration was determined by the Bradford method [40] using bovine serum albumin (BSA) as standard.

### Western blot analysis

Proteins from total cell lysates were resolved by SDS-PAGE in 10% polyacrylamide gels, and transferred by electroblotting to nitrocellulose membranes (Hybond ECL, Amersham Biosciences). Blots were probed for 1 h with monoclonal rat anti-HA (hemagglutinin) antibodies 1/ 500 (Roche), with monoclonal mouse anti-α-tubulin antibodies 1/1,000 (Sigma) or with polyclonal rabbit anti-GDH (glutamate dehydrogenase) antibodies 1/1,000 (GeneTex). The membranes were then washed and incubated for 1 h with goat anti-rat or goat anti-mouse Alexa Fluor^®^ 790 1/25,000, or with goat anti-rabbit Alexa Fluor^®^ 680 1/25,000 (Jackson Immuno Research). Bound conjugate was detected using an Odyssey clx infrared imaging system. Alternatively, after incubation with rat anti-HA primary antibodies, membranes were washed and probed for 1 h with anti-rat horseradish peroxidase conjugate 1/10,000 (Sigma). Bound conjugate was detected by SuperSignal^®^West Pico Chemiluminescent Substrate (Pierce) in conjunction with X-ray film exposure (AGFA GP-BU New Medical).

### Molecular mass determination

The apparent subunit molecular mass of recombinant proteins TcRPE1 and TcRPE2 (GenBank Accession codes ABW88687.1 and ABW88688.1, respectively) was estimated by SDS–PAGE, as described by Weber and Osborn [41]. As a first approach, the native recombinant TcRPE1 and TcRPE2 molecular mass was determined by gel filtration in a Superdex 75 HR 10/30 column (GE Healthcare Amersham Biosciences) with a gel bed of 24 ml. Tris–HCl (50 mM), pH 7.6, containing 500 mM NaCl was used as eluent. Column calibration was performed using as molecular mass standards: cytochrome *c* (12.4 kDa), myoglobin (17 kDa), α-chymotrypsinogen A (25.7 kDa), and bovine serum albumin (66 kDa). Blue Dextran (2000 kDa) was used as a marker of the exclusion volume. Protein samples were prepared in the same elution buffer and centrifuged (20,000 x g at 4°C for 5 min) before injection. Samples and standards (200μl) were resolved at a flow rate of 0.5 ml min^−1^. Similarly, for size exclusion chromatography—light scattering experiments (SEC-FPLC-LS), elution profiles of a Superose 12 column (GE Healthcare Amersham Biosciences) equilibrated at room temperature with 50 mM Tris-HCl buffer, pH 7.6, containing 500 mM NaCl, were recorded following the UV absorption at 280 nm (Jasco UV2075 plus), the enzymatic activity and the multi-angle static light scattering (MASLS) and dynamic light scattering (DLS) signals from in-line modules (Wyatt Technology). Protein samples were prepared in the same elution buffer and centrifuged (20,000 x g at 4°C for 5 min) before injection. Data processing was carried out with the ASTRA software (Wyatt). The samples (100 μl) and the standards were resolved at a flow rate of 0.4 ml min^−1^.

### Homology modeling

The primary sequences of TcRPE1 and TcRPE2 (GenBank Accession codes ABW88687.1 and ABW88688.1, respectively) were obtained from the NCBI protein database and were analyzed using PSI-PRED [42] for the prediction of secondary structure. A Pfam [43] search yielded conserved domains. SCOP [44] analysis was performed to detect domains based on similarity with experimental structures. Local alignments were built using the Basic Local Alignment Search Tool for proteins (BLASTP) [45] accessible at the NCBI-BLAST (http://www.ncbi.nlm.nih.gov/BLAST). Homologous entries were obtained from the RCSB Protein Data Bank (PDB) [46]. At first, template selection criteria were based on three different aspects: (i) extent of identity with the target, (ii) coverage of the target and (iii) crystallographic resolution of the template. This resulted in a short list of 10 possible templates. Then, to choose the best templates, we considered the evolutionary closeness of the targets to the templates. Finally, the putative RPE from *Plasmodium falciparum* (PDB ID: 1TQX) -displaying 48% and 38% sequence identity with TcRPE1 and TcRPE2, respectively- and the putative RPE from *Toxoplasma gondii* (PDB ID: 4NU7) -displaying 50% and 47% sequence identity with TcRPE1 and TcRPE2, respectively- were the templates of choice for the modeling of the monomer of TcRPE1 and TcRPE2. In this analysis we also included as a template the biochemically validated human RPE (PDB ID: 3OVQ) that displays 52% and 47% sequence identity with TcRPE1 and TcRPE2, respectively. The BLAST alignment was further refined by manual curation, and the final sequence alignment file was used as input to Modeller 9.14 [47]. Homology modeling of TcRPEs was performed by running Modeller from a Python terminal. One hundred models for each TcRPE based on each of the three templates (simple homology model) were built. The best model for each set was chosen, according to their DOPE and PDF scores. The best models were evaluated through DOPE profiles construction and different sets of parameters calculated by on-line servers. Finally, a refinement process based in energy minimization of the whole molecule was performed, using the 3DRefine server [48], and the resulting refined models were compared with the starting models to assess their quality improvement. For the refined models of TcRPEs, a specific refinement process for loops was assayed. Neither procedure resulted in significant model improvement. The evaluation of the final models was performed through the analysis of different stereochemical parameters tested by the automatic servers Rampage [49], ProQ [50], ERRAT [51], and QMEAN [52]. Finally, the comparison of the results of the evaluation and validation of the three best final models for each TcRPE was the basis for choosing only one model for each isoenzyme.

### Cloning and over-expression of HATcRPE1, TcRPE1HA, HATcRPE2 and HATcRPE2SHLxAAA in CL Brener clone epimastigotes

Despite many attempts, we were unable to obtain polyclonal antibodies that would allow us to unambiguously discriminate between wild type TcRPEs isoenzymes. Therefore, to assess their subcellular localization we had no choice but to work with the over-expression of tagged TcRPE1 and TcRPE2.

With the aim of generating a HA-tagged construct of TcRPE1 and TcRPE2 for parasite over-expression, the *TcRPE1* and *TcRPE2* ORFS were amplified by PCR from the pGEM^®^-T Easy constructs generated (see above). Primers were designed according to the sequence data obtained from GenBank^™^ database. MluI and BamHI sites were included to facilitate the directional cloning into the expression vector. The sequences of the primers were as follows: sense primer **HATcRPE1FW:**5'- ACGCGT***ATG****TATCCGTATGATGTGCCGGATTATGCG*TTTGATCACCAGGACCGTGG-3', **HATcRPE2FW:** 5'-ACGCGT***ATG****TATCCGTATGATGTGCCGGATTATGCGCGC*AGCTTTGATCATCAGGAC-3' (start bold, MluI underlined and HA tag in italics), **TcRPE1HAFW:** 5'-ACGCGT**ATG**TTTGATCACCAGGACCG-3'; reverse primer **HATcRPE1RV:** 5'-GGATCCTTATTTTGGGGTGAGAGACTTC-3', **HATcRPE2RV:** 5'-GGATCCCTAAAGGTGGGATTTCAAGTGG-3', **TcRPE1HARV:** 5'-GGATCC*TTACGCATAATCCGGCACATCATACGGATA*TTTTGGGGTGAGAGACTTCTTTAC-3' and **HATcRPE2SHLxAAARV**: 5'-GGATCCCTAATGCTGCTGCTTCAAGTGG-3' (BamHI underlined). PCR conditions were as follows: initial denaturation (300 s at 95°C), 25 cycles of denaturation (30 s at 95°C), annealing (30 s at 58°C), elongation (60 s at 68°C) and a final extension step (90 s at 68°C). The PCR products were isolated from a 1% agarose gel, purified by the QIAquick PCR purification kit protocol (Qiagen), and cloned into a pGEM^®^-T Easy vector (Promega). Sequencing of the products was performed by the automated sequencing service provided by MACROGEN (South Korea). The TcRPE1/TcRPE2 HA tagged genes were excised as MluI/BhamHI fragments from the pGEM^®^-T Easy vector, gel purified, and subcloned into the MluI and BamHI sites of the pTcINDEX expression vector [53]. Then *T*. *cruzi* CL-Brener epimastigotes were transfected with circular pLEW13 [53] plasmid and recombinant parasites were selected on 300 μg ml^-1^ G418 (Invivogen). Stably transfected parasites were obtained after eight weeks. Then, CL Brener [pLEW13] epimastigotes were transfected with circular pTcINDEX-HATcRPE1, pTcINDEX-TcRPE1HA, pTcINDEX-HATcRPE2 and pTcINDEX-HATcRPE2SHLxAAA, lacking the DNA cassette that addresses the integration into the non-transcribed ribosomal RNA spacer region. Stably transfected parasites were obtained after eleven weeks, and the selection process was carried out on 300 μg ml^-1^ hygromicin B (Invivogen). To test the capability of the vector to mediate tetracycline-induced expression, an induction assay was performed with tetracycline at a final concentration of 5 μg ml^-1^. The antibiotic was added once to epimastigotes in the early mid-logarithmic growth phase (approximately 2 x 10^7^ parasites ml^-1^). Parasites were grown at 28^◦^C, with shaking. Samples were removed 24, 48, 72, and 96 h later and analyzed by Western blot and fluorescence microscopy. To determine growth for the wild-type and transfected epimastigotes, growth curves were started at 1.2 x 10^7^ cells ml^-1^. Four independent experiments were performed and each time the cells were counted in triplicate daily for 8 days.

### Immunofluorescence assay

Along the logarithmic growth phase, epimastigotes from HATcRPE1, TcRPE1HA, HATcRPE2, HATcRPE2SHLxAAA and pLEW13 lines were collected by centrifugation, washed twice with phosphates-buffered saline (PBS) and fixed in PBS containing 4% p-formaldehyde for 15 min. Parasites were washed twice, resuspended at 2 x10^7^ parasites ml^-1^ in PBS, and attached to polylysine coated glass coverslips for 10 min. Then, coverslips were washed twice with PBS, treated with 25 mM NH_4_Cl for 10 min, and washed again twice with PBS. For permeabilization and blocking, coverslips were then incubated with 2% BSA, 2% normal goat serum and 0.1% saponin in PBS (blocking solution) for 30 min under humidified atmosphere. After that treatment, coverslips were incubated with rat monoclonal anti-HA antibodies (Roche, Basel, Switzerland) and/or mouse polyclonal antibodies against the glycosomal marker *T*.*cruzi* phosphoenolpyruvate carboxykinase (PEPCK) [54], both at a 1/500 dilution, in blocking solution for 1 h under humidified atmosphere and then, washed extensively with PBS. After this procedure, coverslips were incubated with Alexa Fluor 488 conjugated goat anti-mouse secondary antibodies (Invitrogen) and/or Rhodamine Red X Goat anti-rat (Pierce) (1/1,000 diluted in blocking solution) for 1 h in a humidified atmosphere and then washed extensively with PBS. Finally, coverslips were stained and mounted with 4,6-diamidino-2-phenylindole (DAPI) (LifeTechnologies) 1/1,000 diluted in FluorSave (Merck Millipore). Subcellular localization studies were performed using a Nikon Eclipse E600 fluorescence microscope attached to a Sport RT Slider digital camera (model 2.3.1, Diagnostic Instruments, Sterling Heights, MI, USA). Confocal studies were performed with a confocal laser microscope Olympus FV1000 attached to an inverted microscope Olympus IX81 (Melville, NY, USA). Confocal images were acquired in sequential mode with the FluoView (version 3.3, Olympus, Melville, NY, USA). Images were processed in Adobe Photoshop (version 8.0.1; Adobe Systems, San Jose, CA, USA) and pseudo-coloring of images and fluorescence profiles analysis was carried out using the ImageJ software (version 1.43u).

### Statistical analysis

All the assays shown were performed in duplicate or triplicate, and at least three independent experiments were performed. Data are presented as the mean ± SEM or SD as indicated in each particular case. Statistical analysis of the data was carried out using unpaired two-tailed Student’s t test on GraphPad Prism 5 software. The significance of the differences between experimental groups follows the convention: *P < 0.05, **P < 0.005 and ***P < 0.0001.

## Results

### The genome of the CL Brener clone of T.cruzi encodes two RPE isoenzymes

**Fig 1:**
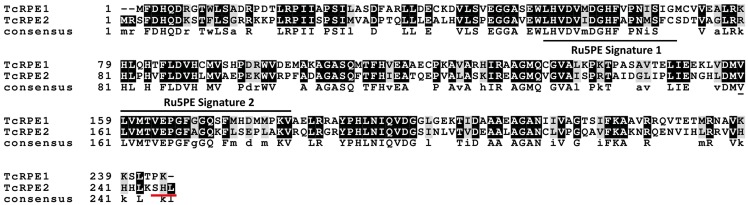
Comparision of amino acid sequences of the RPEs from the CL Brener clone of *T*.*cruzi*. Conservation has been indicated by different tones of grey according to the Boxshade convention (darker grey means more similar residues). At the consensus line, identical residues are represented in uppercase letter and similar residues in lowercase. The PTS glycosomal targeting signal of TcRPE2 is underlined in red and the ribulose 5-phosphate 3’-epimerase family signatures—Ru5PE Signature 1 and Ru5PE Signature 2- are marked with black horizontal lines over and under the letters, respectively.

Two genes encoding ribulose-5-phosphate epimerase from *T*.*cruzi* CL Brener clone were identified: *TcRPE1* (GenBank Accession code: EU075265.1) and *TcRPE2* (GenBank Accession code: EU075266.1). The two isoenzymes of RPE are 54% identical at the amino acid residue level (Fig 1) and one of them (TcRPE2) predicts a PTS1 glycosomal targeting signal (SHL) at the C-terminus. In addition, while in silico predictions -using the ScanProsite tool [55] accessible at the ExPASy: Bioinformatic Resource Portal site (https://www.expasy.org)- yield two different ribulose 5-phosphate 3’-epimerase family signatures (named Ru5PE Signature 1 and 2) in TcRPE1, TcRPE2 presents only one (Ru5PE Signature 2). It is important to notice that these signatures do not correspond to independent substrate binding motifs; both of them contain residues which are part of the active site, and most of these residues are present in both isoenzymes, despite the lack of a complete Signature 1 in TcRPE2 (see the section on “Homology modeling of TcRPE1 and TcRPE2”).

Since *T*.*cruzi* CL Brener strain (TcVI) is a hybrid [56] and it presents several duplicated genes accounting for the existence of isoforms, it is important to emphasize the presence of RPE isoforms in other non-hybrid *T*.*cruzi* strains. Furthermore, the presence of RPE isoforms is not only observed for non-hybrid *T*.*cruzi* strains, but it is also a widespread feature among the different *T*. *cruzi* Discrete Typing Units (DTUs TcI-TcVI) [57]. *T*.*cruzi* CL Brener Esmeraldo-like (TcII) presents two RPE isoforms -TryTrip Gene IDs: TcCLB.510257.30 and TcCLB.507009.30-, *T*.*cruzi* CL Brener Non-Esmeraldo-like (TcIII) also presents two RPE isoforms: TryTrip Gene IDs: TcCLB.509213.70 and TcCLB510667.140. The same occurs with *T*.*cruzi* Dm28c (TcI): TryTrip Gene IDs: TCDM_06074 and TCDM_00120; and with *T*.*cruzi marinkellei* [58] strain B7 (Bat Restricted subspecies): TryTrip Gene IDs: Tc_MARK_8636 and Tc_MARK_4409. This feature is also observed for other protozoan parasites, such as *T*. *brucei* and *Leishmania spp* (see below). The TcRPE1 and TcRPE2 amino acid sequences (GenBank Accession codes ABW88687.1 and ABW88688.1, respectively) were compared with those for the corresponding enzymes from other protozoan parasites, *E*. *coli*, and humans (S1 and S2 Figs). The highest identity: 71.3% for TcRPE1 and 77.7% for TcRPE2 was observed with the *T*. *brucei* putative RPEs (XP823426.1 and CAQ55499.1, respectively), followed by those from *Leishmania major Friedlin*: XP001685917.1 is 55.7% identical to TcRPE1 and XP003722762.1 is 55.9% identical to TcRPE2. A considerable identity level occurs for the RPEs from *Toxoplasma gondii* (4NU7, 50% for TCRPE1 and 47% for TcRPE2), human (3OVQ, 52% for TcRPE1 and 47% for TcRPE2), and *Plasmodium falciparum* (1TQX, 48% for TcRPE1 and 38% for TcRPE2). Finally, a greater divergence was observed when both RPEs were compared with that from *E*.*coli* (3CT7, 34 and 33% identity for TcRPE1 and TcRPE2, respectively).

### Cloning, expression and purification of RPE1 and RPE2 from T.cruzi CL Brener clone

Both TcRPEs were cloned in the bacterial expression plasmid pET-22b(+) and expressed as recombinant C-terminal (His)_6_-tagged proteins in *E*.*coli* BL21 codon Plus (DE3) cells (S3A Fig). The cell free extracts containing the recombinant (His)_6_-tagged proteins were applied to a Ni^2+^ Resin (Invitrogen) for immobilized metal ion affinity chromatography (IMAC) purification (S3B and S3C Fig). Purity of the recombinant RPEs was analyzed by SDS-PAGE followed by Coomassie Blue staining. For both isoenzymes a substantial amount of the protein was expressed as a soluble, active enzyme that could be purified to homogeneity. A striking difference in specific activity between TcRPE1 and TcRPE2—almost 80-100-fold- was observed. Specific activity of the purified recombinant proteins was 52.4 μmol min^-1^mg^-1^ for TcRPE1 and 0.69 μmol min^-1^mg^-1^ for TcRPE2.

### Optimal pH and determination of kinetic parameters

Due to the high specific activity of TcRPE1 we had to use the enzyme highly diluted, but we found that this caused a progressive decay of the enzyme activity if the same diluted sample was used for all determinations. TcRPE1 lost about 40–50% of its initial activity 3 h after dilution. The presence of the substrate Ru5P at a final concentration of 1 mM or the addition of bovine serum albumin (1 to 2 mg ml^-1^) did not prevent this decay (S4 Fig). For this reason, we had to employ this enzyme diluted just before the start of each assay.

**Fig 2:**
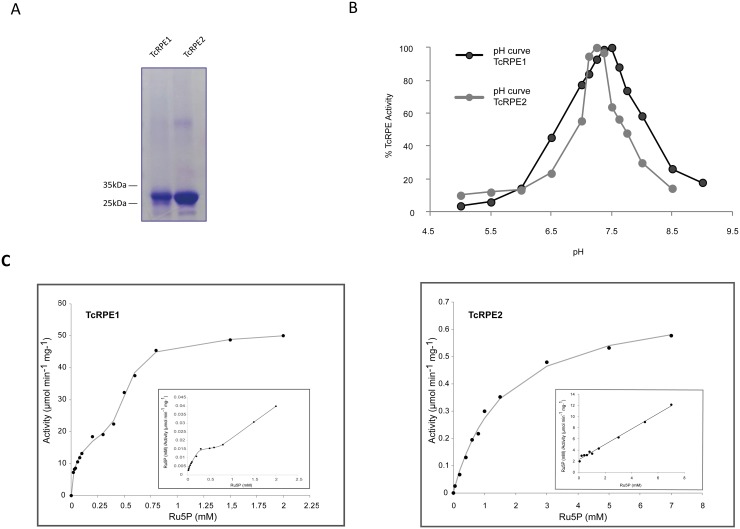
**(A) Recombinant TcRPE1 and TcRPE2 preparations used for enzymatic assays.** SDS-PAGE analysis followed by Coomassie Blue staining to assess the purity of the recombinant proteins TcRPE1 and TcRPE2. **(B) Relative RPE activity as a function of pH for TcRPE1 and TcRPE2.** Acetate, Bis-Tris, Tris-HCl buffer (50 mM each) was used over the complete pH range. Maximal activity was also observed at the same pH values if Tris-HCl or triethanolamine-HCl buffers were used instead. **(C) Plot of RPE specific activity as a function of the concentration of the substrate Ru5P for TcRPE1 and TcRPE2.** While TcRPE2 presents a typical hyperbolic response, TcRPE1 shows a very complex kinetic behavior to increasing concentrations of Ru5P. One of four independent experiments is shown as an example for each isoenzyme. The Hanes-Woolf plot is shown as an inset at the lower right of each panel. The experimental points shown are the mean of two determinations.

The optimal pH for the reaction catalyzed by the purified recombinant TcRPEs (Fig 2A) was determined as described in Materials and Methods. Maximal enzymatic activity was observed at pH 7.25 for TcRPE1 and at pH 7.5 for TcRPE2. We show only the results obtained with acetate, Bis-Tris, Tris-HCl buffer (Fig 2B), since it shows the broadest range of buffer capacity. We also tested Tris-HCl and triethanolamine-HCl at the same concentration. Since in triethanolamine-HCl both enzymes exhibit the same pH maxima and their highest stability, this buffer was chosen for all the ensuing activity assays aimed at determining the kinetic parameters.

Surprisingly, both isoenzymes differ markedly in regard to their kinetic behavior (Fig 2C). TcRPE1 showed a very complex kinetic pattern to increasing concentrations of Ru5P, displaying a biphasic curve, suggesting the coexistence of -at least- two kinetically different molecular forms. The linearity of the reaction with time (initial velocity) in all zones of the curves was checked, thus discarding the possibility of an experimental artifact. When the experiment was repeated several times by using different batches of the recombinant TcRPE1, the general pattern was consistently observed, but with a shift in the transition zone between both phases, which might be explained by different proportions of -at least- two kinetically different oligomeric species (see next Section) comprising each batch of recombinant enzyme. Although efforts to find a mathematical expression to calculate the kinetic parameters corresponding to the two phases of the curves were done, reproducible results could not be obtained, and therefore kinetic constants for TcRPE1 are not presented.

In contrast, TcRPE2 showed a typical hyperbolic response to increasing concentrations of Ru5P. After non-linear regression, the values of the kinetic parameters for this isoenzyme were determined at pH 7.5, by fitting TcRPE2 data from four independent experiments to the Michaelis-Menten equation, as described in Materials and Methods. We measured a K_M_ value of 2.21 ± 0.23 mM, a kcat value of 0.32 ± 0.02 s^-1^, and a kcat/ K_M_ of 0.16 ± 0.02 s^-1^ mM^-1^. The K_M_ value obtained for TcRPE2 was similar to that obtained for the recombinant cobalt metalloform RPE from *E*.*coli*, with a K_M_ value of 2.40 ± 0.40 mM [12].

Although we cannot compare the kinetic constants for both isoenzymes, inspection of the graphs in Fig 2C shows that TcRPE1 has a much higher specific activity than TcRPE2, and suggests that at least the kinetic component detected at the lower substrate concentrations must have also an affinity for Ru5P considerably higher than that for TcRPE2.

To test if the TcRPEs were metalloenzymes, various divalent metals were tested for their ability to activate these enzymes *in vitro*. None of the eight cations assayed (Ca^2+^, Co^2+^, Cu^2+^, Fe^2+^, Mn^2+^, Mg^2+^, Ni^2+^, Zn^2+^) at a concentration of 1 mM was able to activate TcRPE1 or TcRPE2 above background levels (S5 Fig). In addition, purified TcRPE1 was pre-incubated for 1 h in the presence of different chelating agents (5 to 10 mM EDTA, 5 to 10 mM EGTA or 1 mM o-phenanthroline). Even after many independent replicates of the assay, inconsistent results were obtained. Since the method we used for RPE activity determination involves a magnesium-dependent TKT as a coupled enzyme, we had to work with TcRPE1 highly diluted after its preincubation with chelating agents, in order to prevent any inhibition of the coupled enzyme. Given that TcRPE2 shows low specific activity, we were not able to test the effect of chelating agents on the activity of this isoenzyme. Although these results suggest that recombinant TcRPE1 does not bear an exposed divalent metal cation essential for enzymatic activity, the aforementioned experimental limitations prevented us to reach a definite answer to this issue.

Finally, the treatment with 1 to 5 mM DTT or with 10 mM cysteine, and the treatment with 5 to 10 μM H_2_O_2_ (S6 Fig) brought about no effect on the activity of either enzyme, suggesting that TcRPEs do not bear thiol groups essential for their activity. This finding is in good agreement with the absence of any conserved cysteine residue in the multiple sequence alignment analysis (S1, S2 and S8 Figs).

### Oligomeric composition of the recombinant TcRPE1 and TcRPE2 from T.cruzi

The subunit size of the recombinant TcRPE1 and TcRPE2 enzymes -as estimated by SDS PAGE- was 27 and 27.7 kDa, respectively (S7 Fig), values that fall in good agreement with those predicted by the theoretical molecular weight computing tool of the Expasy web server [59].

**Fig 3:**
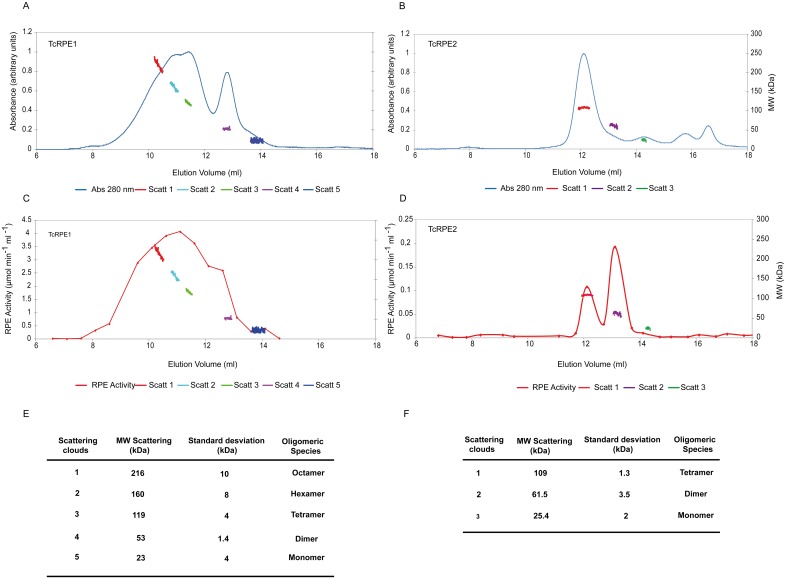
Each TcRPE displays a complex oligomeric composition. Panels **A**, **C** and **B**, **D** correspond to TcRPE1 and TcRPE2, respectively. Absorbance at 280 (thin blue line), molecular mass in kDa estimated by LS (clouds points of different color) and RPE activity (thin red line). Panels E and F summarize in tabular form the different oligomeric states found for TcRPE1 and TcRPE2, respectively.

To determine the native molecular weight for both recombinant TcRPEs, we initially carried out size exclusion chromatography (SEC) experiments. Given that more than one active peak eluted for each TcRPEs, and as the assignment of each peak to a defined molecular weight was unclear, we decided to run a SEC experiment using a Superose 12 column, equipped with an on-line light scattering detector (SEC-FPLC-LS) (see Materials and Methods). This approach is much more reliable and accurate for the determination of molecular masses than SEC alone [60,61], because it is able to provide independent and absolute (i.e. calibration-free) values of molecular mass. In addition, measuring the absorbance at 280 nm alongside each chromatographic run allowed us to yield a profile where for each eluted peak an associated molecular mass cloud could be estimated from the scattering signal. Surprisingly, through its application we found that both TcRPEs present multiple oligomeric species. This trait is unique among all the RPEs studied so far [1–9,14], that present only one oligomeric species apart from the monomer. LS reveals the presence of species in solution with molecular masses of 23, 53, 119, 160 and 216 kDa for TcRPE1 (Fig 3A) (corresponding to monomer, dimer, tetramer, hexamer and octamer, respectively) and molecular masses of 25, 62 and 109 kDa for TcRPE2 (corresponding to monomer, dimer and tetramer, respectively) (Fig 3B). Remarkably, all the oligomeric forms found for each TcRPE result from associations of an even number of molecules. This new finding could be explained if dimers act as the minimal assembly units giving rise to the larger oligomeric structures observed. This interpretation is consistent with previous reports for some RPEs [9,10,14]. The different fractions eluted from the Superose 12 column were collected, and their activity was measured (Fig 3C and 3D). We found activity associated to each peak fractions showing scattering. However, for both isoenzymes the monomers presented only marginal activity. The TcRPE1 isoenzyme shows maximal enzymatic activity for the larger oligomeric species (119–160 kDa). By contrast, TcRPE2 does not show higher activity for the larger oligomers (the maximum occurs for the dimeric form of 61.5 kDa). The TcRPE1 and TcRPE2 isoenzymes not only organize themselves into different sets of oligomeric species -with TcRPE1 showing a higher proportion of oligomeric species rich in larger oligomers- but there is also a difference between isoenzymes regarding the distribution of activity associated to each oligomer. The more complex oligomeric profile and the RPE activity distribution among the different oligomers observed for TcRPE1 might be linked to the complex kinetic behavior of this isoform. However, since the different oligomeric species of TcRPEs might be involved in a dynamic equilibrium mixture that will necessarily be affected by dilution, unambiguous assignment of specific activity for any of these oligomeric forms is not straightforward.

### Homology modeling of TcRPE1 and TcRPE2

To determine the tertiary structure of each TcRPE isoenzyme, non-automatic homology modeling was performed using Modeller, with the aid of a Python programming interface, so as to control each step along the process. In order to look for template candidates, the RCSB (Protein Data Bank) database was searched. The amino acid sequences extracted were compared (S8 Fig). Basing our decision criterion on different features of the templates -as described in Materials and Methods- the same 3 templates were chosen for each TcRPE: the putative RPE from *Plasmodium falciparum* (PDB ID: 1TQX), the putative RPE from *Toxoplasma gondii* (PDB ID: 4NU7), and the biochemically validated human RPE (PDB ID: 3OVQ). Three sets of 100 models were built for each TcRPE based on each chosen template. The best model for each set was selected, evaluated, refined and re-evaluated -as described in Materials and Methods- and final refined models were compared to choose only one for each TcRPE (Tables 1 and 2). Results from these comparisons showed that the best models for TcRPE1 and TcRPE2 are those constructed with 1TQX and 3OVQ templates,respectively.

**Table 1:** Evaluation of the best three molecular models for TcRPE1.

TcRPE1										
Templates	Identity (%)	Coverage (%)	Ramachandran (%)			ERRAT	ProQ (LG score)	Verify 3D (%)	Q-MEAN (Z-score)	RMSD (Å)
			Favorable	Allowed	Disallowed					
**1TQX**	48	92	98.3	4.5	1.7	93.6	4.4	95.5	-0.32	0.49 (215 paired Cα atoms)
**4NU7**	50	91	92.1	5.8	2.1	85.2	3.6	99.6	-1.13	1.67 (216 paired Cα atoms)
**3OVQ**	52	89	94.2	3.7	2.1	80.1	3.9	93.4	-0.93	0.78 (208 paired Cα atoms)

Comparison among the best three molecular models for TcRPE1 built with different templates. The best final model of choice is shaded in yellow.

**Table 2:** Evaluation of the best three molecular models for TcRPE2.

TcRPE2										
Templates	Identity (%)	Coverage (%)	Ramachandran (%)			ERRAT	ProQ (LG-score)	Verify 3D (%)	Q-MEAN (Z-score)	RMSD (Å)
			Favorable	Allowed	Disallowed					
**1TQX**	38	91	94.7	4.1	1.2	82.8	3.9	82.1	-1.02	2.05 (217 paired Cα atoms)
**4NU7**	47	88	92.2	4.9	2.9	77.2	3.9	93.3	-1.45	1.24 (216 paired Cα atoms)
**3OVQ**	47	87	95.5	4.5	0	90.1	4.6	83	-0.69	1.01 (209 paired Cα atoms)

Comparison among the best three molecular models for TcRPE2 built with different templates. The final model of choice for is shaded in yellow.

**Fig 4:**
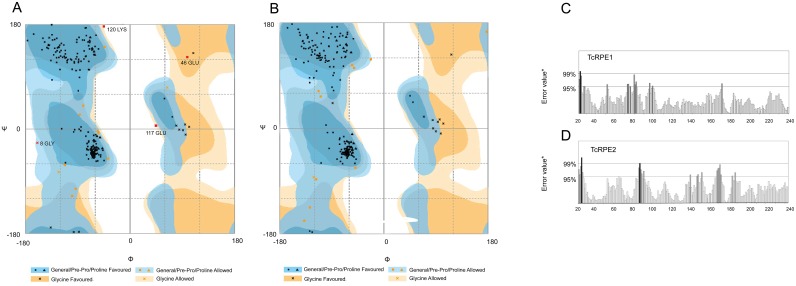
Overall Ramachandran and Errat plots for the refined models of TcRPE1 (A, C) and TcRPE2 (B, D).

**Fig 5:**
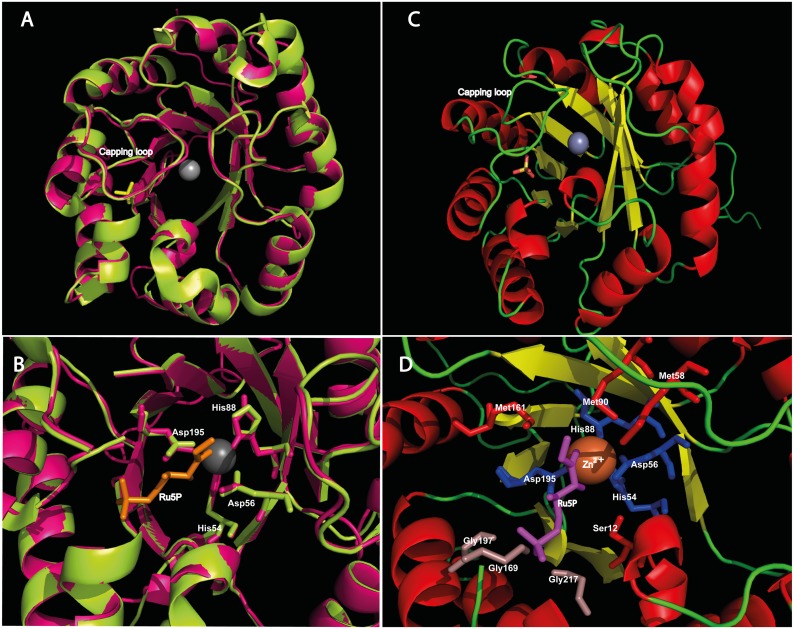
**(A) Ribbon representation of the superimposition between the model for TcRPE1 (in magenta) and its template 1TQX (in lemon green).** The coordinated zinc ion is shown as a grey sphere and the bound sulfate ion is represented in yellow sticks. **(B)** Zoom view of the superimposition of the active site, showing the catalytic tetrad in stick representation. Ru5P is represented in orange sticks. **(C)** Ribbon depiction of the overall structure of the TcRPE1 model: the central β-sheet core is shown in yellow, the αβ loops in green, and the α-helices in red. **(D)** Zoom view on the active site of the model for TcRPE1, where the catalytic tetrad is shown in blue, and residues involved in substrate docking are shown in grey and red sticks. Ru5P is painted in purple.

The structural quality control analysis of the final 3D models chosen for TcRPE1 and TcRPE2 was carried out using the following servers: ProQ [50], Rampage [49], ERRAT [51], Q-MEAN [52] and the Verify 3D program [48] installed in Structural Analysis and Verification Server tools (SAVES). The Ramachandran plot produced for the refined model of TcRPE1 by Rampage showed 98.3% residues in favorable regions, 4.5% in allowed regions and 1.7% in disallowed regions. Correspondingly, for the refined model of TcRPE2, 95.5% residues occur in favorable regions, 4.5% in allowed regions, and no residue was found in disallowed regions (Fig 4A and 4B). This analysis indicates that the backbone dihedral angles psi and phi in each protein model are reasonably accurate. ERRAT plots indicate overall quality factors of 93.6 and 90.1 (very good quality) for the TcRPE1 and TcRPE2 models, respectively (Fig 4C and 4D). In addition, the models were also analyzed with the ProQ server, a neural network based quality predictor, obtaining LG scores of 4.4 for TcRPE1 and 4.6 for TcRPE2. Thus, both meet the criteria for very good models. Furthermore, for both TcRPE models the quality of the fit of each amino acid sequence to the environment dictated by the protein fold was evaluated using Verify3D, obtaining 95.5 and 83% of the residues with an average 3D-1D score > = 0.2 for TcRPE1 and TcRPE2, respectively, indicating good compatibility between the atomic model (3D) and the target amino acid sequence (1D). In general, the folding energy of proteins shows negative values, a fact consistent with the stability and native character of the molecules. We evaluated this energy using the Q-MEAN server, obtaining z-scores of -0.32 for TcRPE1 and -0.69 for TcRPE2. For both models this energy remains negative for all amino acid residues, indicating a consistently high level of quality. All in all, the analysis of these sets of parameters let us be confident on the reliability of the models created for TcRPE1 and TcRPE2. The spatial superimposition between the models for TcRPE1 and TcRPE2 and their corresponding templates 1TQX and 3OVQ shows that both models bear close similarity to their templates, with an overall RMSD value of 0.49 Å for TcRPE1 (for 215 paired Cα atoms Fig 5A) and a RMSD value of 1.01 Å (for TcRPE2 for 220 paired Cα atoms S9 Fig). Given that this homology modeling exercise yields similar results for each TcRPE, only the results for the TcRPE1 isoenzyme are shown and further discussed. The overall structure of the TcRPE1 monomer folds into a single α/β domain shaped as a variant of a classical TIM-barrel (Fig 5C). A central eight-stranded parallel β-sheet makes up the cylindrical core of the barrel. This nucleus is surrounded by eight α-helices, inserted between consecutive strands and connected with them by αβ loops. Flexible loops spanning residues G60 to S67 and E168 to P172 are conserved between the model and the template. It has been reported that they fold over the active site, and are well positioned to sequester reaction intermediates during catalysis [9]. The active site of the TcRPE1 template structure (*P*. *falciparum* RPE) shows two bound ligands: a zinc and a sulfate ion, which are embedded in the model structure. Superimposition of the binding pocket (Fig 5B) shows that the canonic catalytic tetrad (H54, D56, H88, D195) is fully conserved. According to information available for the template that was used for modeling TcRPE1, these residues participate in divalent zinc ion coordination at the active site and have also been reported to do so in other known RPEs [8,9,11]. These divalent ions are putatively involved in catalysis, stabilizing a 2, 3-enediolate intermediate [8]. In addition, the presence of amino acid residues that were previously reported as important for substrate docking, such as G169, G197 and G217, that had been implicated in the constriction of the active site [9]; M58, M90 and M161, involved in polar cushioning to help stabilize the 2, 3-enediolate intermediate [11,16]; and S12, associated to a hydrogen bonded network important for the relay of charge in the enzyme-substrate binary complex [11], are present and well positioned in TcRPE1 model (Fig 5D). With the exception of M58, all the residues mentioned above are also present in TcRPE2 model. The superimposition of the binding pocket of both TcRPE1 and TcRPE2 models shows that they are also found in essentially identical positions in the active site (residue specific RMSD < 2Å), with the exception of S12 which displays a residue specific RMSD of 11.07 Å (S10 Fig).

### Over-expression and subcellular localization

**Fig 6:**
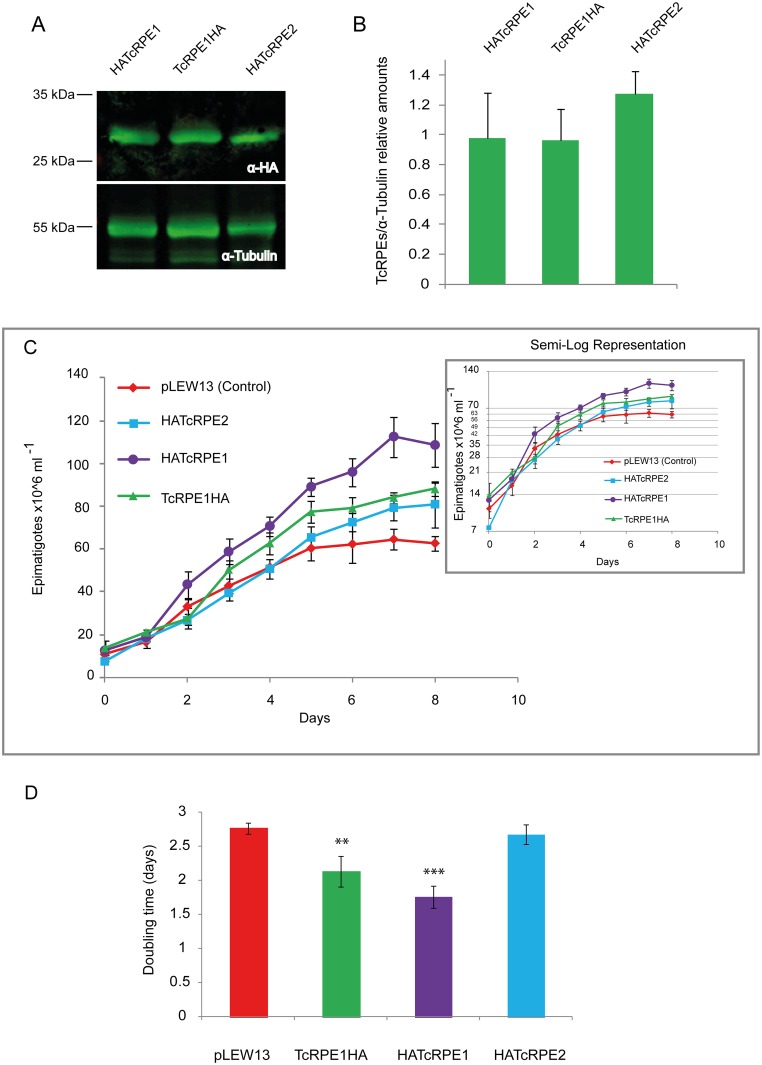
HATcRPE1 and TcRPE1HA over-expression enhances the growth of *T*. *cruzi* CL Brener epimastigotes. **(A)** Equal amounts of total cell free extracts from HATcRPE1, TcRPE1HA, and HATcRPE2 CL Brener [pLEW13] lines after 72h induction with 5 μg/ ml tetracycline were loaded on SDS PAGE followed by Western blot analysis using rat anti-HA monoclonal antibodies (α-HA) and mouse anti-tubulin antibodies (α-tubulin). **(B)** The intensity of the HATcRPE1, TcRPE1HA and HATcRPE2 bands was quantified in three independent experiments and normalized to α-tubulin intensity. The bar graph represents the mean ± SEM of the relative intensity of the bands. **(C)** Growth curves of pLEW13 (control), HATcRPE1, TcRPE1HA and HATcRPE2 strains under tetracycline induction. One of four independent experiments is shown as an example. The semi-log growth is shown as an inset. Black bars represent the SD mesured by counting cell number daily by triplicate. **(D)** Doubling times of the HATcRPE1, TcRPE1HA and HATcRPE2 strains were estimated from four independent experiments. The bar graph represents the mean ± SEM of the doubling time.

The intracellular localization of TcRPE1 and TcRPE2 was studied in cultured epimastigotes of *T*.*cruzi* CL Brener clone by immunofluorescence. While TcRPE2 presents a PTS1 glycosomal targeting signal (SHL) at the C-terminus [62], the TcRPE1 amino acid sequence lacks conspicuous organelle targeting signals, suggesting a possible cytoplasmic fate. To reveal the localization of these proteins, both TcRPEs were cloned in the tetracycline-inducible expression vector pTcINDEX [53] and expressed as recombinant HA-tagged proteins in CL Brener pLEW13 epimastigotes by means of an induction assay with tetracycline. Samples for Western blot and immunofluorescence analysis were taken 72 h after tetracycline induction. The TcRPE1 isoenzyme was HA-tagged at its N (HATcRPE1 line) or C-terminus (TcRPE1HA line), while TcRPE2 was only HA-tagged at its N-terminus (HATcRPE2 line) to prevent any effect on its PTS1 glycosomal targeting signal (SHL) at the C-terminus. As expected, episomal over-expression resulted in production of a ~30 kDa protein for all the lines, in good agreement with the predicted value for both TcRPEs (taking into account the added 9 amino acid residues from the HA tag), as detected by Western blot analysis using an anti-HA antibody (Fig 6A). No differences on the over-expression were observed when all the transfected lines were compared by means of a semi-quantification analysis of the Western blot images (Fig 6B). For HATcRPE1 and TcRPE1HA lines, there was a considerable leak in the absence of tetracycline, but -given our goals-, this did not represent an obstacle (S11 Fig). After the heterologous expression of the different HA tagged constructions was confirmed, we proceeded to investigate the growth of the transfected epimastigotes *in vitro*. pLEW13 (control), HATcRPE1, TcRPE1HA and HATcRPE2 parasites were grown in BHT medium and monitored for 8 days until the stationary phase had been reached (Fig 6C and S12 Fig). The over-expression of HATcRPE1 and TcRPE1HA did significantly reduce the parasite doubling time *in vitro* (doubling times of 1.76 ± 0.08 days, p< 0.0001 unpaired *t* test and 2.14 ± 0.11 days, p< 0.005 unpaired *t* test, respectively), compared with the pLEW13 control (2.77 ± 0.04 days). In contrast, the over-expression of HATcRPE2 (doubling time of 2.68 ± 0.07) did not significantly modify the parasite doubling time observed under normal conditions (Fig 6D).

**Fig 7:**
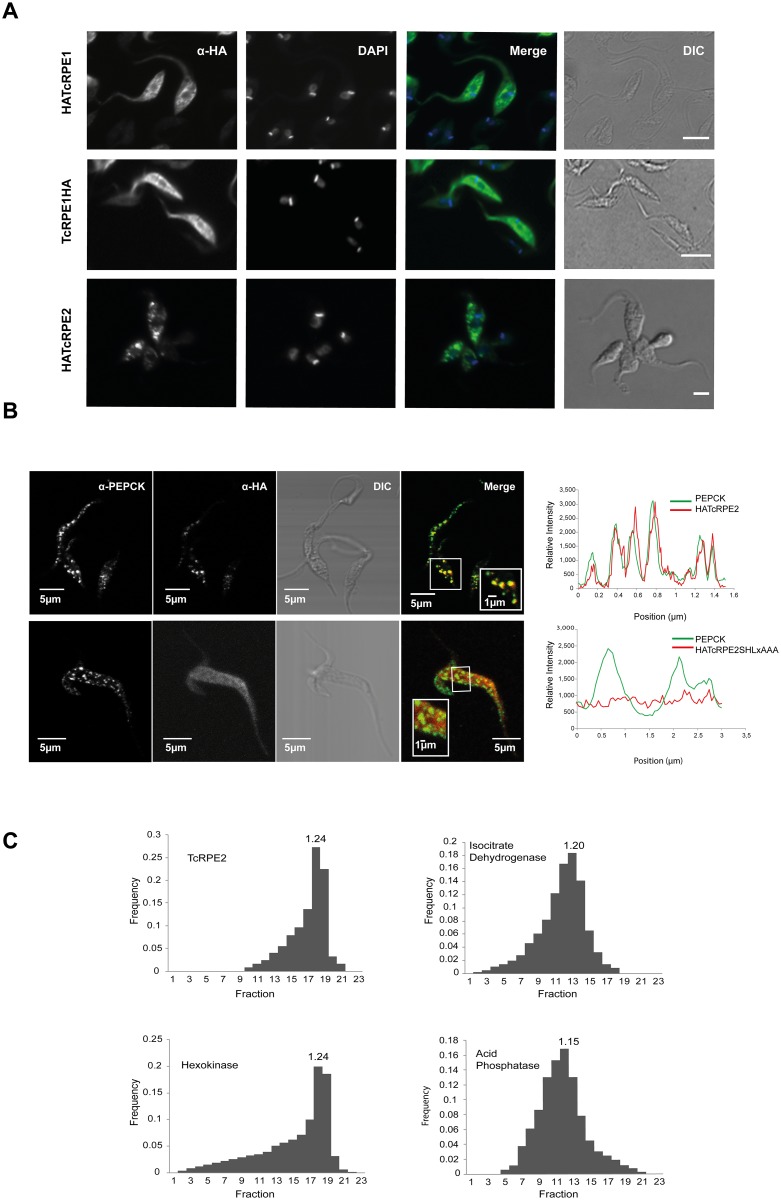
Subcellular localization of the two RPE isoenzymes. **(A)** Immunofluorescence assay of epimastigotes from HATcRPE1, TcRPE1HA, and HATcRPE2 CL Brener [pLEW13] lines after 72 h induction with tetracycline. From left to right: Parasites immunostained with rat anti-HA antibodies, nuclei and kinetoplasts (DAPI), merge image, and cell bodies visualized by interference contrast (DIC). The bar equals 5 μm. **(B)** Confocal images showing HATcRPE2, HATcRPE2SHLxAAA and PEPCK co-localization in CL Brener [pLEW13] lines after 72 h induction with tetracycline. From left to right: parasites immunostained with mouse anti-PEPCK polyclonal antibodies, parasites immunostained with rat anti-HA antibodies, cell bodies visualized by interference contrast (DIC), merge image, and line profiles of fluorescense relative intensity as a function of position for HATcRPE2 and HATcRPE2SHLxAAA (red line) and PEPCK (green line). The bar equals 5 μm. **(C)** Sucrose linear density gradient centrifugation of small granule fraction obtained from intact wild type epimastigotes of *T*.*cruzi* CL Brener clone. Plot of frequency (calculated as described in Materials and Methods) as a function of the fraction number ordered by increasing density. From left to right and from top to bottom, the activity of the following enzymes was assayed: TcRPE2; isocitrate dehydrogenase, a mitochondrial marker; hexokinase, a glycosomal marker, and acid phosphatase, a Golgi apparatus marker. Equilibrium densities of the fraction exhibiting maximal activity is indicated in each panel and expressed in g ml^-1^.

Fluorescent microscopy analysis shows that TcRPE1 is mainly localized in the cytosol, regardless of where the HA tag is placed. Instead, TcRPE2 shows a particulate localization pattern, in agreement with the presence of the PTS1 signal at its C-terminus (Fig 7A). In addition, fluorescent confocal analysis shows co-localization between TcRPE2 and the glycosomal marker phosphoenolpyruvate carboxykinase (PEPCK), suggesting that TcRPE2 is indeed a glycosomal enzyme. Moreover, this co-localization is lost when the same analysis is run with the HATcRPE2SHLxAAA mutant bearing the substitution of the SHL putative glycosomal targeting signal for AAA, showing that the subcellular localization of TcRPE2 is dependent on its targeting signal (Fig 7B). The over-expression of HATcRPE2SHLxAAA did significantly enhance the parasite doubling time *in vitro* (3.21 ± 0.07 days, p< 0.005 unpaired *t* test) compared with the pLEW13 control, even when its over-expression was lower than that observed for HATcRPE2 (S13 Fig). This phenomenon is in agreement with the observations made for *T*.*brucei* [63] where the over-expression in bloodstream trypanosomes of the glycosomal enzyme PGKC (phosphoglycerate kinase C), lacking the final 5 amino acids which are responsible for glycosomal targeting, resulted in a toxic effect that causes cell death.

Furthermore, to ascertain the subcellular localization of natural TcRPEs in epimastigotes, we performed a sucrose linear density gradient centrifugation with the SG fraction obtained by subcellular fractionation of wild type CL Brener clone parasites (as described in Materials and Methods). Our results show that TcRPE2 presents an activity peak coincident with that observed for the activity peak of the glycosomal marker hexokinase (Fig 7C), evidence that also agrees with the glycosomal localization expected for this enzyme.

## Discussion

At variance with all the other PPP enzymes from *T*.*cruzi*, TcRPE presents two isoenzymes sharing 53% identity with each other: TcRPE1 and TcRPE2. In this study, both isoenzymes were expressed as His-tagged recombinant proteins, which resulted functionally active for the interconversion of Ru5P into Xu5P. Despite high sequence similarity, TcRPE1 shows a very different specific activity: 80-100-fold greater than TcRPE2, and a strikingly different kinetic behavior. Whereas TcRPE2 shows a typical hyperbolic response to increasing concentrations of Ru5P, TcRPE1 shows a complex kinetic pattern, displaying a biphasic curve, suggesting the presence of -at least- two different kinetic components. This kinetic pattern is similar to the one previously reported for the malic enzyme II of *T*.*cruzi* [64] and for anthranilate synthetase from *Salmonella typhimurium* [65], but it has never been reported for any of the enzymes of the PPP pathway in *T*.*cruzi*, *T*.*brucei*, *Leishmania* or other trypanosomatids. For the TcRPE1 isoenzyme, circumstantial evidence points to the null effect of divalent cations on its activity. However, a definitive answer to this point will require further analysis. In the case of RPEs from other sources for which crystallographic structures have been obtained, some of them present a divalent cation [8–11] at the active site, whereas others do not [14,16].

The tertiary structure for both TcRPEs was studied by a non-automatic homology modeling procedure that included control checkpoints along the modeling process, ultimately leading to the generation of very accurate structures. As expected for proteins sharing a high identity with each other (more than 40% according to established criteria), both TcRPEs constitute single domains exhibiting the classical α/β TIM-barrel fold, as shown for other enzymes with RPE activity. For both isoenzymes there is only one active site per monomer subunit. With regard to the architecture of the active site, we found that most of the important amino acid residues for catalysis and substrate binding are present in both isoenzymes, with the sole exception of M58 that is absent in the TcRPE2 active site. It is noteworthy that the triad of M residues (M58, M90, and M161) has been reported to participate in the stabilization of the reaction intermediate [11, 16], and therefore the lack of one of them might affect catalysis. The superimposition of the binding pocket of both TcRPE1 and TcRPE2 models shows that catalytically essential amino acids adopt identical orientations in space (a residue specific RMSD < 2Å for all of them), with the exception of S12 displaying a residue specific RMSD of 11.07 Å. Interestingly, this residue has been proposed to participate in the relay of charge in the enzyme-substrate binding complex [11]. The subtle differences mentioned above in regard to the organization of the active site might contribute to explain the striking difference in specific activity between the two isoenzymes, and the apparently considerably lower affinity of TcRPE2 for the substrate Ru5P.

An approach to the quaternary arrangement of these enzymes carried out by light scattering reveals a distinctive feature of TcRPEs. Both isoenzymes present a mixture of various oligomeric species made up of an even number of units, probably pointing to the dimer as the minimal entity capable of exerting catalysis. This multiplicity of oligomeric species has not been reported for any other RPE studied so far [1–9,14]. Suggestively, involvement of these oligomers in a dynamic equilibrium might be linked to the progressive loss of stability observed for TcRPE1 as the enzyme is diluted. Equilibrium perturbation by dilution of the equilibrium existing among different TcRPE1 oligomeric species might bring about a change in oligomeric composition and -consequently- in specific activity. Moreover, this feature might play a role in explaining the complex kinetic pattern observed for the TcRPE1 isoenzyme. In this scenario, TcRPE1 shows a maximum of enzymatic activity for large oligomeric species (119–160 kDa) whereas for TcRPE2 the maximal activity is centered around the dimeric form (61.5 kDa). This fact might help explain why increased complexity in oligomeric composition in the case of TcRPE1 might find a correlate with increased complexity in the kinetic pattern. Although further investigation will be necessary to unravel the physiological significance of these findings, this trait differentiates TcRPEs from RPEs from all the other sources studied so far and it might bear implications for the regulation of TcRPE activity. Different cellular conditions could promote a change in TcRPEs oligomeric composition, allowing the regulation of these isoenzymes under different physiological circumstances, in a fashion similar to that reported for the 6-phosphogluconate dehydrogenase of *T*.*brucei* [66], another enzyme of the pentose phosphate pathway.

Regarding the subcellular compartmentalization of TcRPEs we found that in *T*. *cruzi* epimastigotes, whereas TcRPE1 adopts a cytosolic location, TcRPE2 shows a perfect co-localization with the glycosomal marker PEPCK, pointing to the effective migration of this protein to glycosomes, as expected from its PTS1 C-terminal signal. Furthermore, the subcellular localization of TcRPE2 is absolutely dependent on the presence of its targeting signal, since the over-expression of a truncated mutant -HATcRPE2SHLxAAA- lacking the putative glycosomal targeting signal, not only remains in the cytosol, but also negatively affects parasite growth, extending its doubling time *in vitro*. Collectively, this evidence points to the physiological importance of TcRPE2 compartmentalization, as has been previously shown for the case of phosphoglycerate kinase C from *T*.*brucei* [63]. On the other hand, the over-expression of HATcRPE1 and TcRPE1HA shortens the epimastigotes doubling time *in vitro*, whereas the over-expression of HATcRPE2 does not significantly alter the parasite growth curve measured under normal conditions. The positive effect in epimastigotes growth as a consequence of TcRPE1 over-expression might be attributed to an increased metabolic flux towards the non-oxidative branch of the PPP generated by TcRPE1 over-expression, enabling a better satisfaction of nucleotide biosynthesis requirements for cell proliferation, through the incremental production of ribose 5-phosphate.

To the best of our knowledge, TcRPE2 is the first reported PPP isoenzyme that is exclusively localized in glycosomes in *T*.*cruzi* epimastigotes, where most of PPP enzymes share only a minor glycosomal component [19]. For *T*.*brucei* [67,68] and *Leishmania spp* [35,69,70] only a partial glycosomal component for some of the PPP enzymes has been previously reported.

Taken together, our findings on the kinetics, structure and localization of both TcRPEs from *T*.*cruzi* CL Brener clone describe striking differences between both isoenzymes. The very unique features of these enzymes might add a further regulation layer of importance for the physiology of the parasite, specifically for the physiological role of the PPP in the cytosol and the glycosome. However, there is, so far as we know, no information about the role of the pathway in these two locations in *T*. *cruzi*. The enzymes of the PPP are encoded in the genomes of *T*. *cruzi*, *T*. *brucei* and *Leishmania spp*, and all of them are expressed in all parasite stages, both in the mammalian host and in the insect vector, with one notorious exception: RPE and TKT are not expressed in the bloodstream trypomastigotes of *T*. *brucei*. Reference [71] reviews the information on the PPP enzymes in the three trypanosomatids, and the possibility that some of them might become targets for chemotherapy. The oxidative branch of the PPP has been shown to be functional in *T*. *cruzi* epimastigotes [19] and in *L*. *mexicana* promastigotes [35], and most probably is also functional in all the other parasite stages, as well as in *T*. *brucei*, since it is essential for the generation of NADPH, required both to protect the parasites against oxidative stress and for biosynthetic processes, and also to provide ribose 5-phosphate for nucleotide synthesis. It is remarkable that, although the non-oxidative branch cannot be operative in bloodstream trypomastigotes of *T*. *brucei*, ribose phosphate isomerase is present, and has been recently shown, by RNAi experiments, to be required for growth *in vitro* and for the bloodstream forms infectivity in mice [34].

Further studies will be required to assess the functionality of the whole non-oxidative branch of the PPP in Trypanosomatids. In the case of *T*. *cruzi*, the evaluation of the essentiality of the different enzymes will be difficult to determine, since it lacks the RNAi gene silencing mechanism, and gene knockout is difficult in this parasite.

## Supporting information

S1 Fig Primary structure analysis of T.cruzi RPE1. The predicted amino acid sequences of TcRPE1 (ABW88687.1), *T*.*brucei* RPE1 (XP823426.1) *L*.*major* Friedlin RPE (XP001685917.1), *T*.*gondii* RPE (4NU7), Human RPE (3OVQ), *P*. *falciparum* RPE (1TQX) and *E*.*coli* RPE (3CT7) were aligned using the Clustal Omega multiple alignment program. Conservation has been indicated by different tones of grey according to the Boxshade convention (darker grey means more similar residues). At the consensus line, identical residues are represented in uppercase letter and similar residues in lowercase. Amino acid residues involved in catalysis are annotated with 1. The sequences corresponding to the ribulose-phosphate-3-epimerase (Ru5PE) family signature 1 and 2 are underlined.
(TIF)

S2 Fig Primary structure analysis of T.cruzi RPE2. The predicted amino acid sequences of TcRPE2 (ABW88688.1), *T*.*brucei* RPE2 (CAQ55499.1) *L*.*major* Friedlin RPE2 (XP003722762.1), *T*.*gondii* RPE (4NU7), Human RPE (3OVQ), *P*.*falciparum* RPE (1TQX) and *E*.*coli* RPE (3CT7) were aligned using the Clustal Omega multiple align program. Conservation has been indicated by different tones of grey according to Boxshade convention (darker grey means more similar residues). At the consensus line, identical residues are represented in uppercase letter and similar residues in lowercase. Amino acid residues involved in catalysis are annotated with 1. The sequence corresponding to the Ru5PE family signature 2, is underlined.
(TIF)

S3 Fig Expression and purification of the two RPE isoforms. **(A)** SDS-PAGE analysis of the expression of the two RPE isoforms (Coomassie Blue staining). SDS-PAGE analysis to assess the purity of the recombinant proteins TcRPE1 **(B)** and TcRPE2 **(C)**, along the different fractions resulting from the IMAC purification (Coomassie Blue staining). The abbreviations Sol and Insol mean supernatant and pellet, respectively.
(TIF)

S4 Fig Dilution of TcRPE1 isoform showed a negative effect over the enzyme stability in time. TcRPE1 was diluted 500-fold in the absence (darker grey) or in the presence (light grey) of 1 mM Ru5P and it was incubated during 3h at room temperature. Aliquots were taken after 20, 60, 120 and 190 minutes, and activity measurements were performed. One of three independent experiments is shown as an example. The black bars represent the mean ± SD obtained after measure the activity in duplicated for each of the treatments.
(TIF)

S5 Fig TcRPE1 (A) and TcRPE2 (B) are not activated by the addition of divalent cations. None of the eight cations assayed (1 mM Ca^2+^, 1 mM Co^2+^, 1 mM Cu^2+^, 1mM Fe^2+^, 1 mM Mn^2+^, 1 mM Mg^2+^, 1 mM Ni^2+^, and 1 mM Zn^2+^) was able to activate recombinant TcRPEs above background levels. One of three independent experiments is shown as an example. The black bars represent the mean ± SD obtained after measure the activity in duplicated for each of the treatments.
(TIF)

S6 Fig TcRPE1 (A) and TcRPE2 (B) do not bear thiol groups essential for their activity. None of the agents assayed (5 mM DTT, 10 mM cysteine or 10 mM H_2_O_2_) was able to inhibit recombinant TcRPEs under background levels. One of three independent experiments is shown as an example. The black bars represent the mean ± SD obtained after measure the activity in duplicated for each of the treatments.
(TIF)

S7 Fig Monomer molecular mass estimated by denaturing gel electrophoresis (SDS-PAGE). Left panel SDS-PAGE followed by Coomassie Blue staining, showing TcRPE1 and TcRPE2. Differents amounts of the recombinant enzymes were used, namely 3, 1.5, and 0.75 μg for TcRPE1, and 0.85, 1.7, 3.4, and 6.8 μg for TcRPE2. Right panel: plot of the logarithm of molecular mass as a function of relative mobility for several molecular weight markers and for the TcRPE enzymes.
(TIF)

S8 Fig Multiple amino acid sequence alignment (MSA) of the TcRPE orthologs deposited in PDB. Amino acid sequences of *Trypanosoma cruzi* RPE1 (ABW88687.1), *Trypanosoma cruzi* RPE2 (ABW88688.1), Human RPE (3OVQ), Rice RPE (1H1Y), *Plasmodium falciparum* RPE (1TQX), *Toxoplasma gondii* RPE (4NU7), *Streptococcus pyogenes* RPE (2FLI), *Francisella tularensis* RPE (3INP), *Synechocystis* RPE (1TQJ), potato chloroplast RPE (1RPX), *E*. *coli* RPE (3CT7) and *Haemophilus somnus* RPE (3CU2) were aligned using the Clustal Omega multiple alignment program. Conservation has been indicated by different tones of grey according to the Boxshade convention (darker grey means more similar residues). At the consensus line identical residues are represented in uppercase letter and similar residues in lowercase. Amino acid residues directly involved in catalysis are annotated with 1, those involved in substrate docking with 3, and those belonging to the capping loop with 2.
(TIF)

S9 Fig Ribbon representation of the superimposition between the model of TcRPE2 (in blue) and its template 3OVQ (in grey). (TIF)

S10 Fig Amino acid residues important for catalysis and substrate docking found in essentially identical positions in the active site of TcRPE1 and TcRPE2 models. Superimposition of the active sites of TcRPE1 and TcRPE2 models. Important amino acid residues for catalysis and substrate docking are shown in red sticks for TcRPE1 and in green sticks for TcRPE2. Ribulose 5-phosphate is shown as yellow sticks. The S12 amino acid residue is not shown to attain clear visualization of the active site residues.
(TIF)

S11 Fig Inducible over-expression of the two TcRPE isoforms. Western blot of cell free extracts from HATcRPE1, TcRPE1HA, and HATcRPE2 CL Brener [pLEW13] lines after 72 h induction with tetracycline, with rat anti-HA antibodies. Different amounts of induced and non induced (control) parasites -ranging from 2 to 20 millions- were loaded to attain clear visualization.
(TIF)

S12 Fig Growth curves of pLEW13 (control), HATcRPE1, TcRPE1HA and HATcRPE2 strains under tetracycline induction. Parasites were grown in BHT medium and followed for 8 days until the stationary phase was reached. Four independent experiments performed for each transfected line are shown.
(TIF)

S13 Fig Inducible over-expression and growth curves of HATcRPE2SHLxAAA. **(A)** Western blot of cell free extracts from HATcRPE2, HATcRPE2SHLxAAA lines after 72 h induction with tetracycline, with rat anti-HA antibodies and rabbit anti-GDH antibodies as loading control.**(B)** The intensity of the HATcRPE2 and HATcRPE2SHLxAAA bands was quantified from three independent experiments and normalized to α-GDH intensity. The bar graph represents the mean ± SEM of the relative intensity of the bands.**(C)** Gowth curves of HATcRPE2SHLxAAA strain under tetracycline induction. Parasites were grown in BHT medium and followed for 10 days until the stationary phase was reached. Three independent experiments were performed, named Curve #1–3. **(D)** Doubling times of the pLEW13, HATcRPE2 and HATcRPE2SHLxAAA lines were calculated from three independent experiments. The bar graph represents the mean ± SEM of the doubing time.
(TIF)
