# Supplementary figures and images for: Structure, kinetic characterization and subcellular localization of the two ribulose 5-phosphate epimerase isoenzymes from Trypanosoma cruzi
Source: PLoS One. 2017 Feb 16;12(2):e0172405. doi: 10.1371/journal.pone.0172405 (PMC5312968; doi:10.1371/journal.pone.0172405)

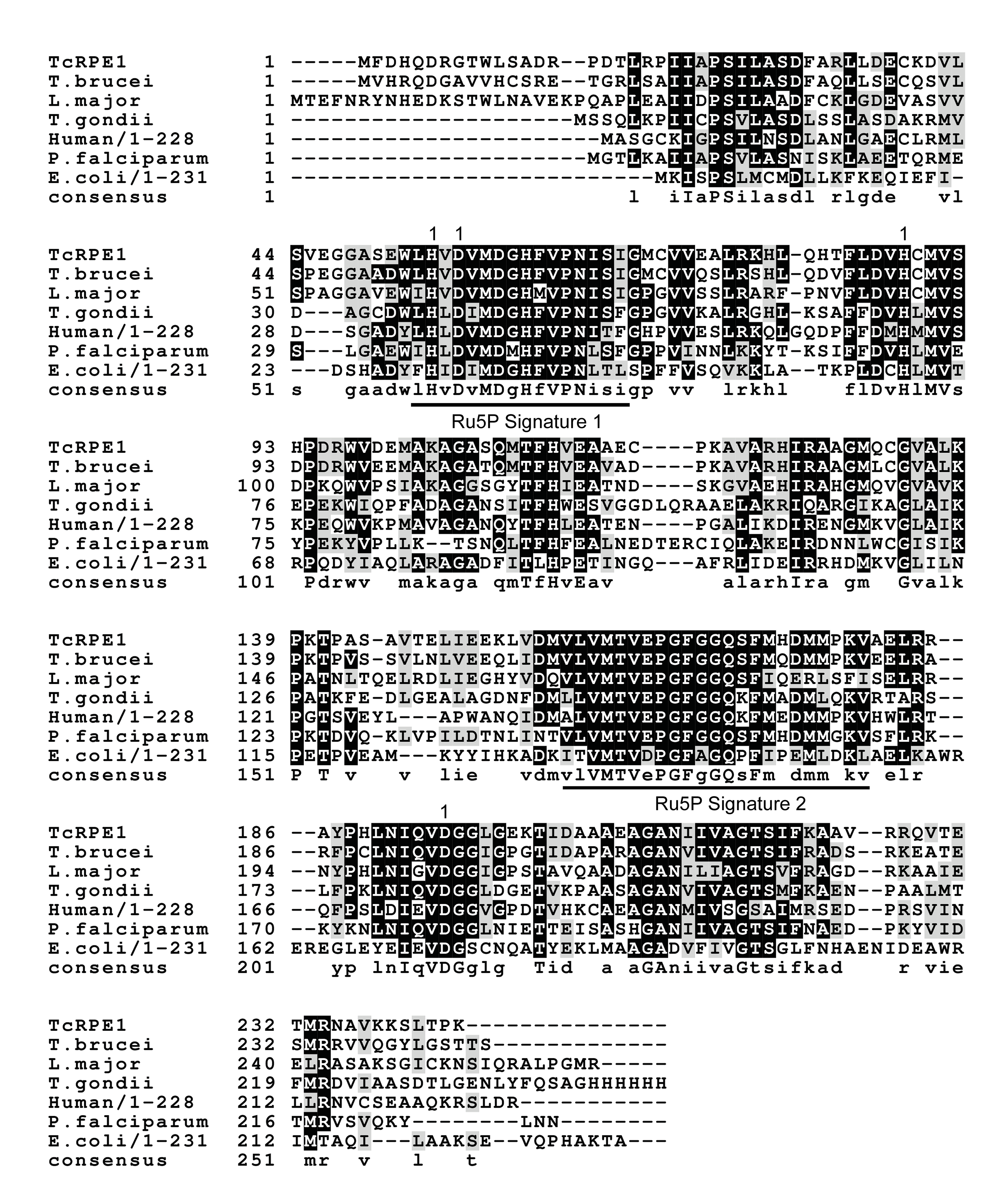

Supplement: S1 Fig — The predicted amino acid sequences of TcRPE1 (ABW88687.1), T.brucei RPE1 (XP823426.1) L.major Friedlin RPE (XP001685917.1), T.gondii RPE (4NU7), Human RPE (3OVQ), P. falciparum RPE (1TQX) and E.coli RPE (3CT7) were aligned using the Clustal Omega multiple alignment program. Conservation has been indicated by different tones of grey according to the Boxshade convention (darker grey means more similar residues). At the consensus line, identical residues are represented in uppercase letter and similar residues in lowercase. Amino acid residues involved in catalysis are annotated with 1. The sequences corresponding to the ribulose-phosphate-3-epimerase (Ru5PE) family signature 1 and 2 are underlined. (TIF) [file pone.0172405.s001.tif]

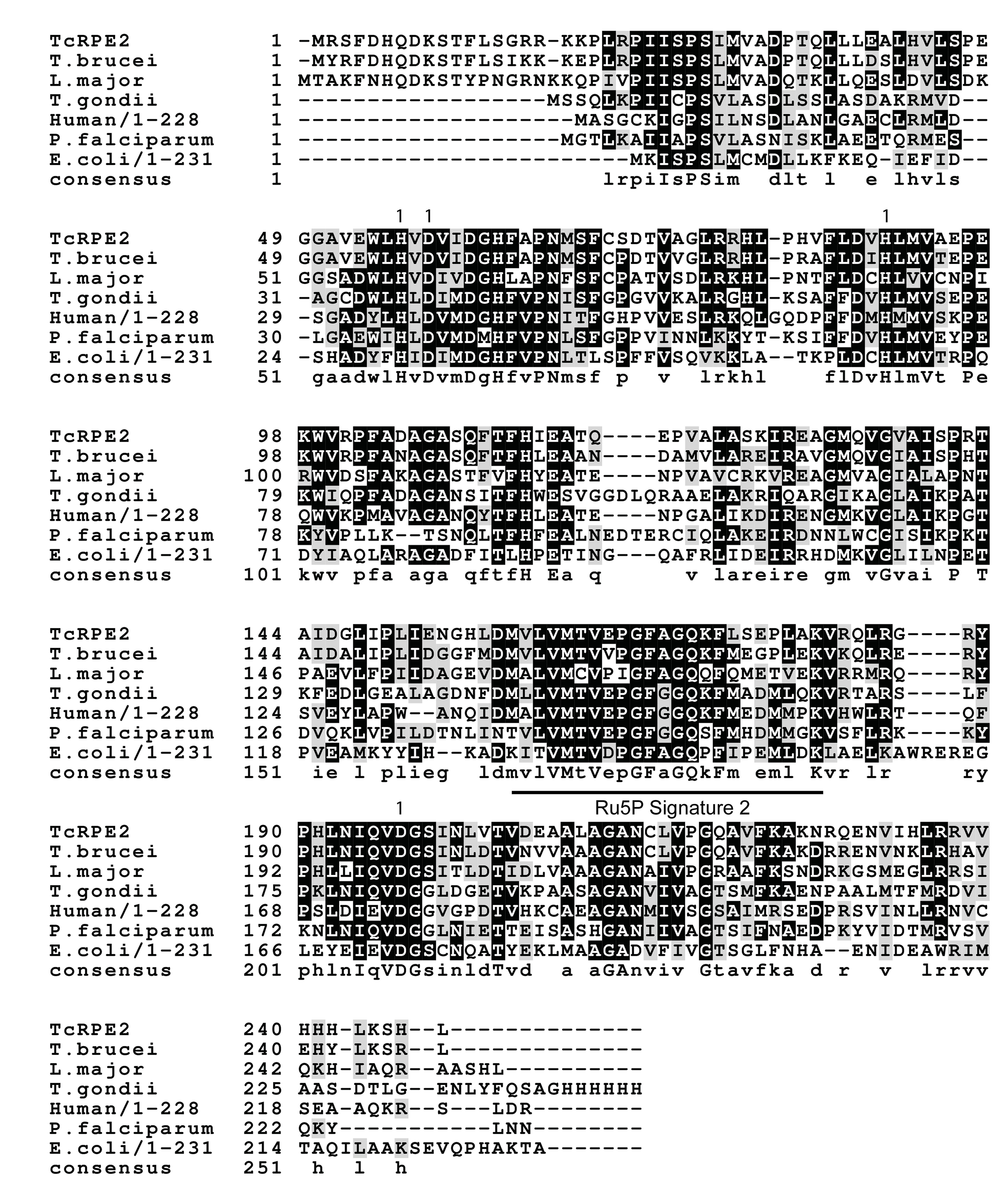

Supplement: S2 Fig — The predicted amino acid sequences of TcRPE2 (ABW88688.1), T.brucei RPE2 (CAQ55499.1) L.major Friedlin RPE2 (XP003722762.1), T.gondii RPE (4NU7), Human RPE (3OVQ), P.falciparum RPE (1TQX) and E.coli RPE (3CT7) were aligned using the Clustal Omega multiple align program. Conservation has been indicated by different tones of grey according to Boxshade convention (darker grey means more similar residues). At the consensus line, identical residues are represented in uppercase letter and similar residues in lowercase. Amino acid residues involved in catalysis are annotated with 1. The sequence corresponding to the Ru5PE family signature 2, is underlined. (TIF) [file pone.0172405.s002.tif]

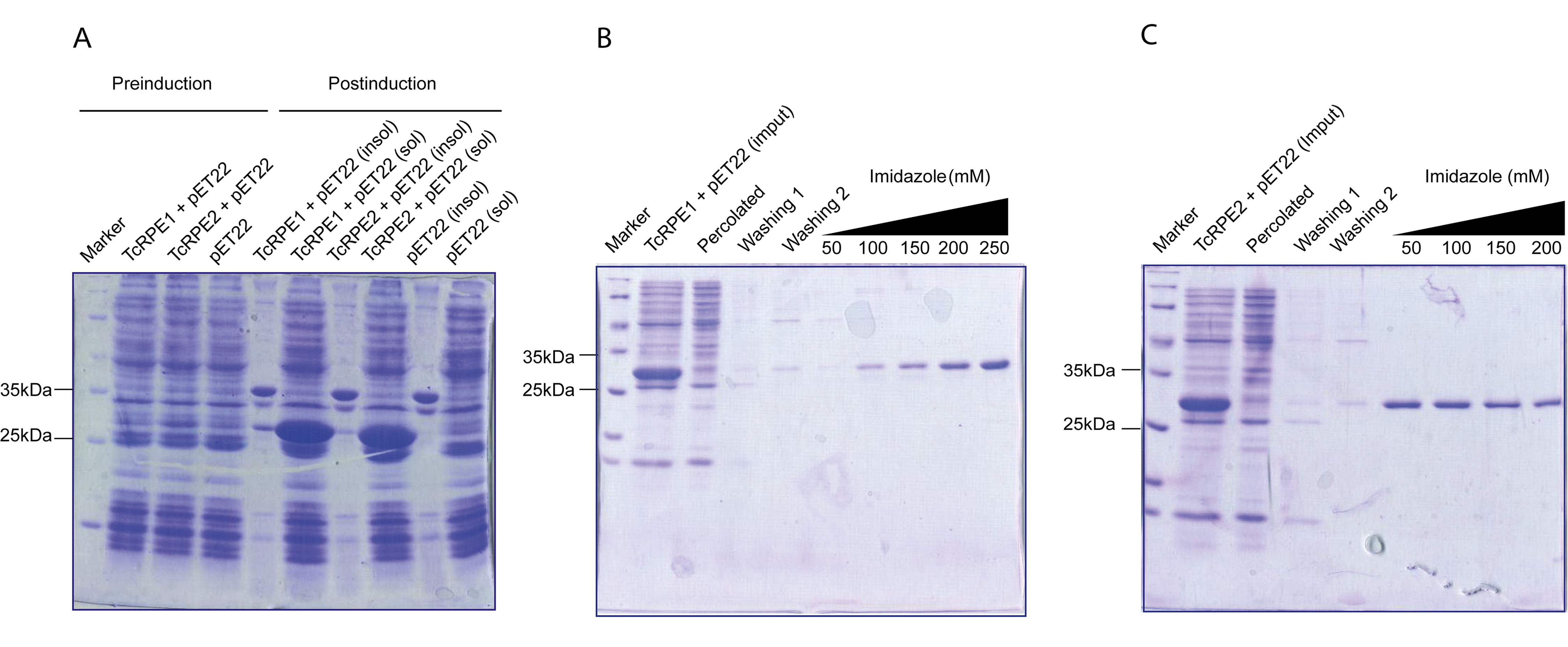

Supplement: S3 Fig — (A) SDS-PAGE analysis of the expression of the two RPE isoforms (Coomassie Blue staining). SDS-PAGE analysis to assess the purity of the recombinant proteins TcRPE1 (B) and TcRPE2 (C), along the different fractions resulting from the IMAC purification (Coomassie Blue staining). The abbreviations Sol and Insol mean supernatant and pellet, respectively. (TIF) [file pone.0172405.s003.tif]

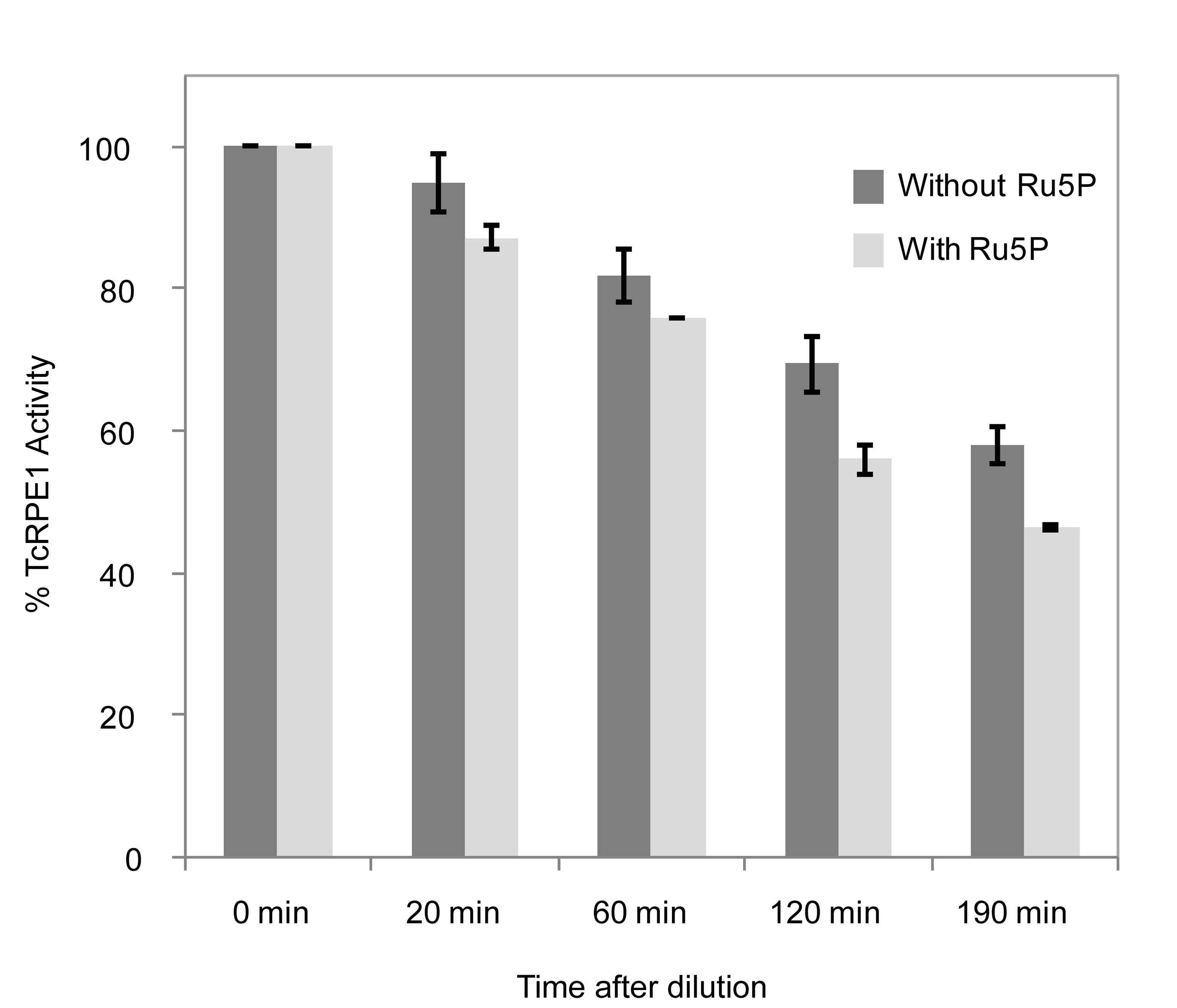

Supplement: S4 Fig — TcRPE1 was diluted 500-fold in the absence (darker grey) or in the presence (light grey) of 1 mM Ru5P and it was incubated during 3h at room temperature. Aliquots were taken after 20, 60, 120 and 190 minutes, and activity measurements were performed. One of three independent experiments is shown as an example. The black bars represent the mean ± SD obtained after measure the activity in duplicated for each of the treatments. (TIF) [file pone.0172405.s004.tif]

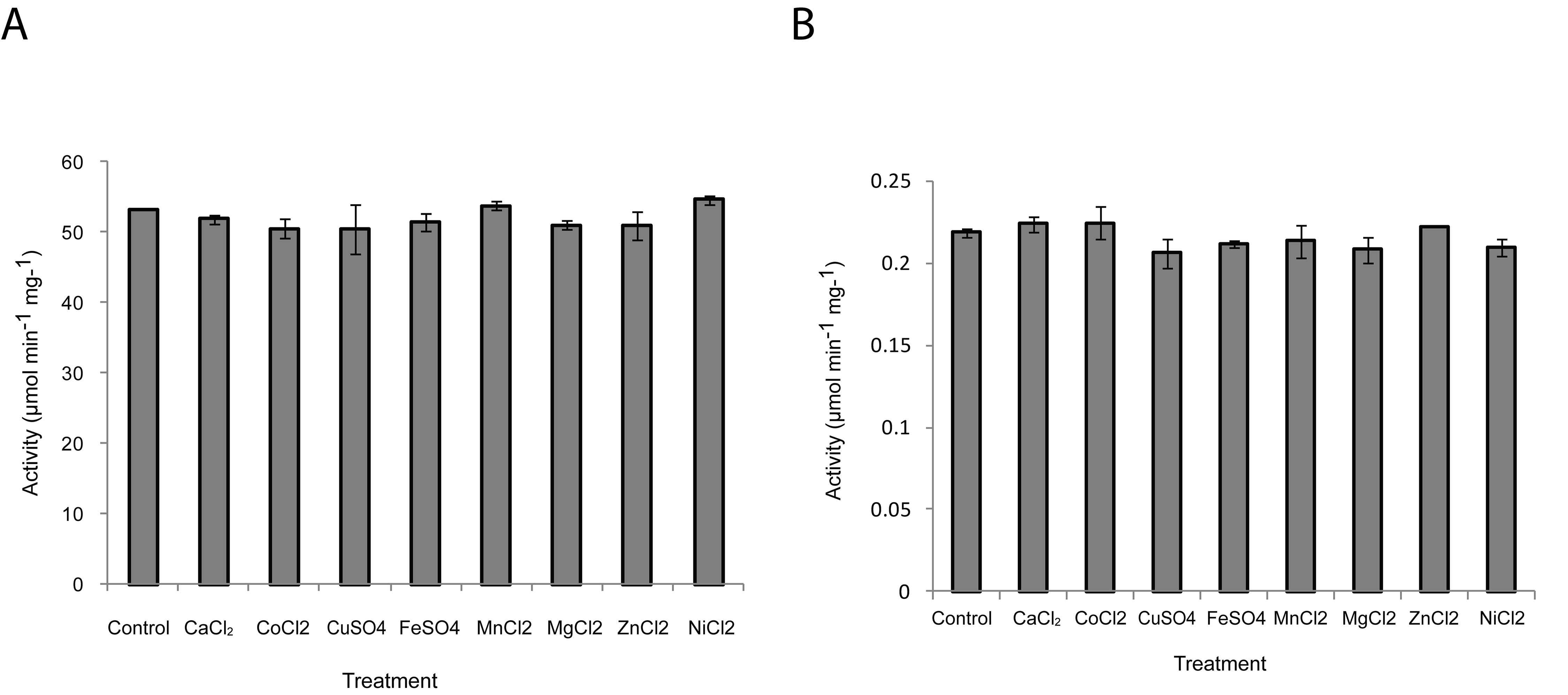

Supplement: S5 Fig — None of the eight cations assayed (1 mM Ca2+, 1 mM Co2+, 1 mM Cu2+, 1mM Fe2+, 1 mM Mn2+, 1 mM Mg2+, 1 mM Ni2+, and 1 mM Zn2+) was able to activate recombinant TcRPEs above background levels. One of three independent experiments is shown as an example. The black bars represent the mean ± SD obtained after measure the activity in duplicated for each of the treatments. (TIF) [file pone.0172405.s005.tif]

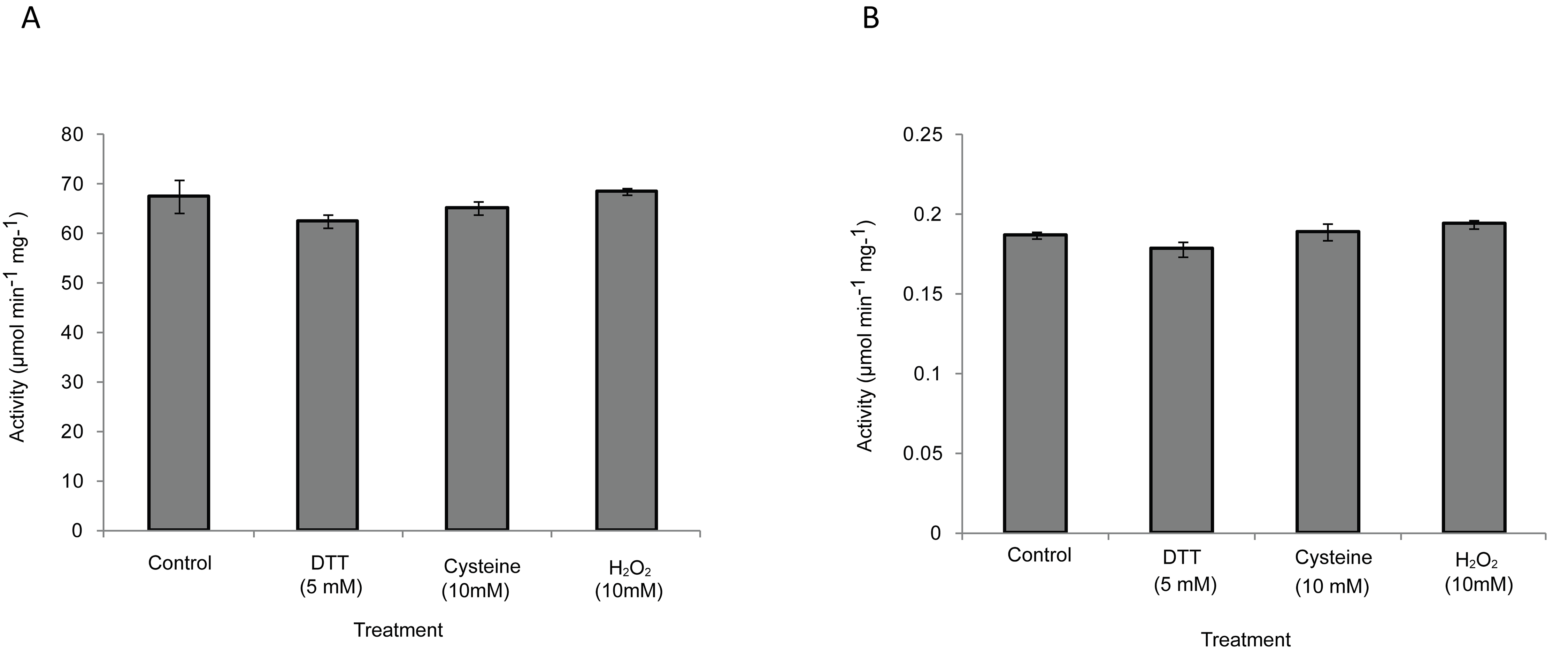

Supplement: S6 Fig — None of the agents assayed (5 mM DTT, 10 mM cysteine or 10 mM H2O2) was able to inhibit recombinant TcRPEs under background levels. One of three independent experiments is shown as an example. The black bars represent the mean ± SD obtained after measure the activity in duplicated for each of the treatments. (TIF) [file pone.0172405.s006.tif]

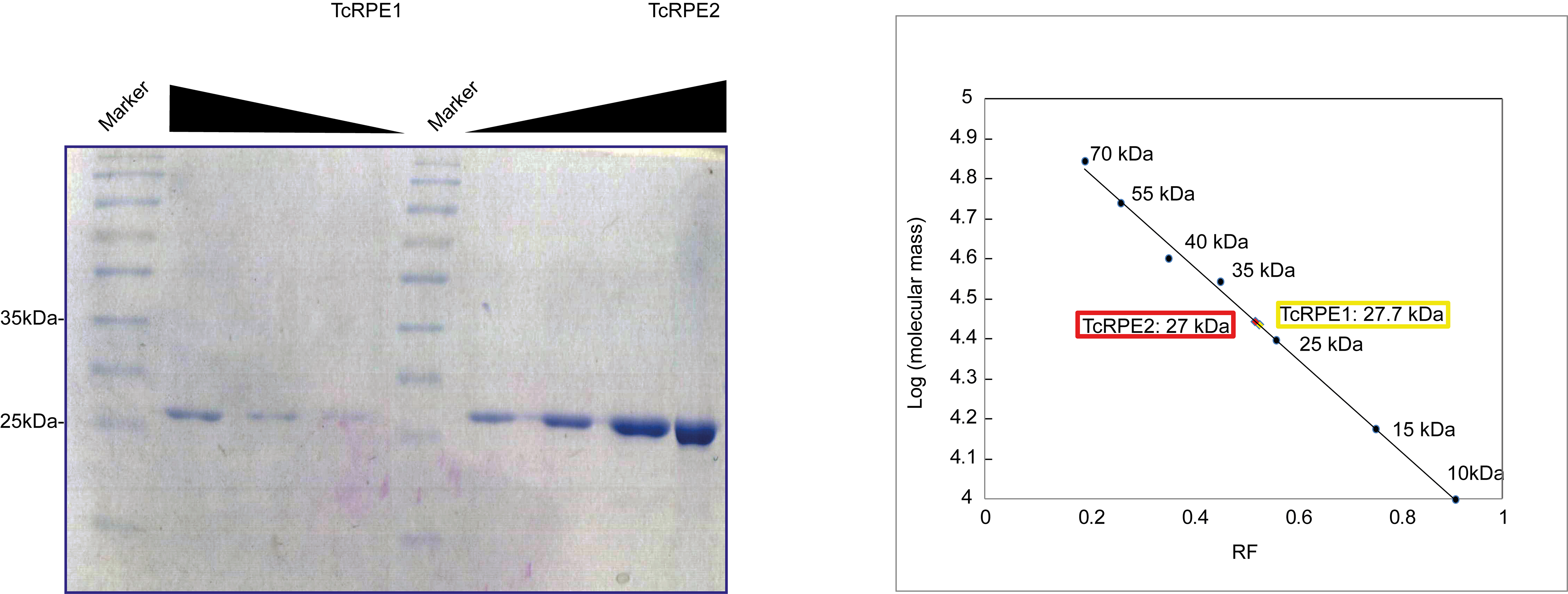

Supplement: S7 Fig — Left panel SDS-PAGE followed by Coomassie Blue staining, showing TcRPE1 and TcRPE2. Differents amounts of the recombinant enzymes were used, namely 3, 1.5, and 0.75 μg for TcRPE1, and 0.85, 1.7, 3.4, and 6.8 μg for TcRPE2. Right panel: plot of the logarithm of molecular mass as a function of relative mobility for several molecular weight markers and for the TcRPE enzymes. (TIF) [file pone.0172405.s007.tif]

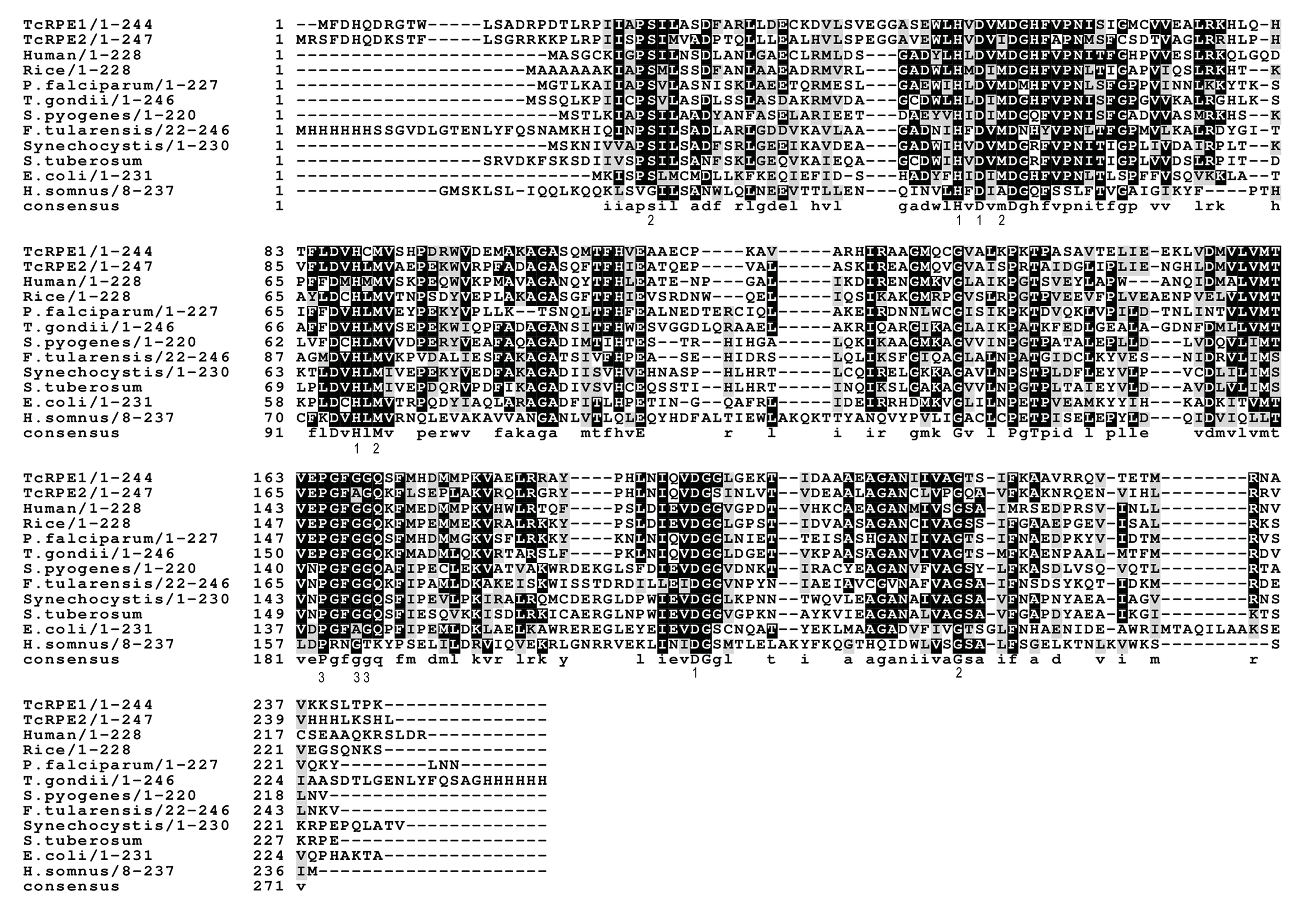

Supplement: S8 Fig — Amino acid sequences of Trypanosoma cruzi RPE1 (ABW88687.1), Trypanosoma cruzi RPE2 (ABW88688.1), Human RPE (3OVQ), Rice RPE (1H1Y), Plasmodium falciparum RPE (1TQX), Toxoplasma gondii RPE (4NU7), Streptococcus pyogenes RPE (2FLI), Francisella tularensis RPE (3INP), Synechocystis RPE (1TQJ), potato chloroplast RPE (1RPX), E. coli RPE (3CT7) and Haemophilus somnus RPE (3CU2) were aligned using the Clustal Omega multiple alignment program. Conservation has been indicated by different tones of grey according to the Boxshade convention (darker grey means more similar residues). At the consensus line identical residues are represented in uppercase letter and similar residues in lowercase. Amino acid residues directly involved in catalysis are annotated with 1, those involved in substrate docking with 3, and those belonging to the capping loop with 2. (TIF) [file pone.0172405.s008.tif]

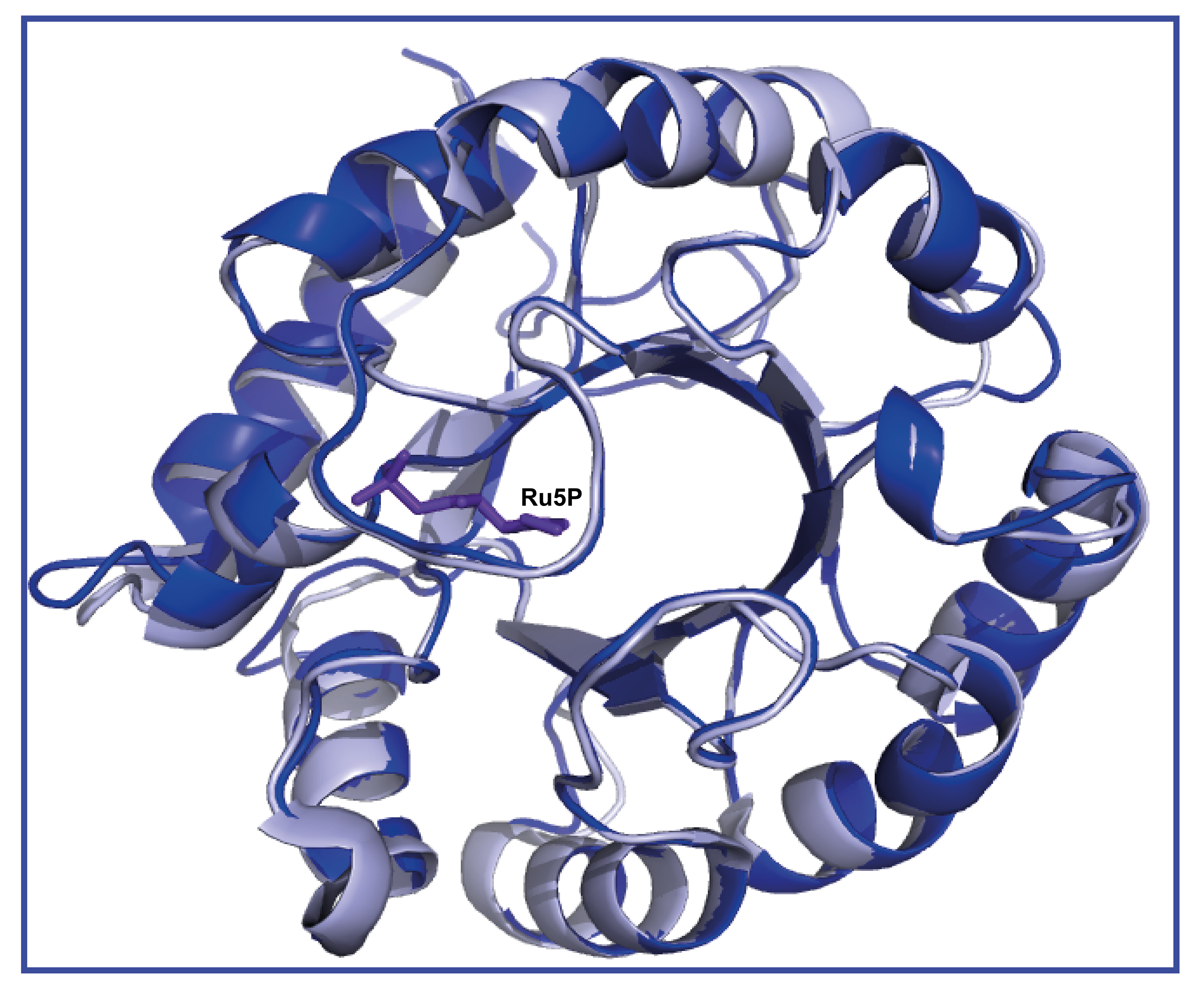

Supplement: S9 Fig — (TIF) [file pone.0172405.s009.tif]

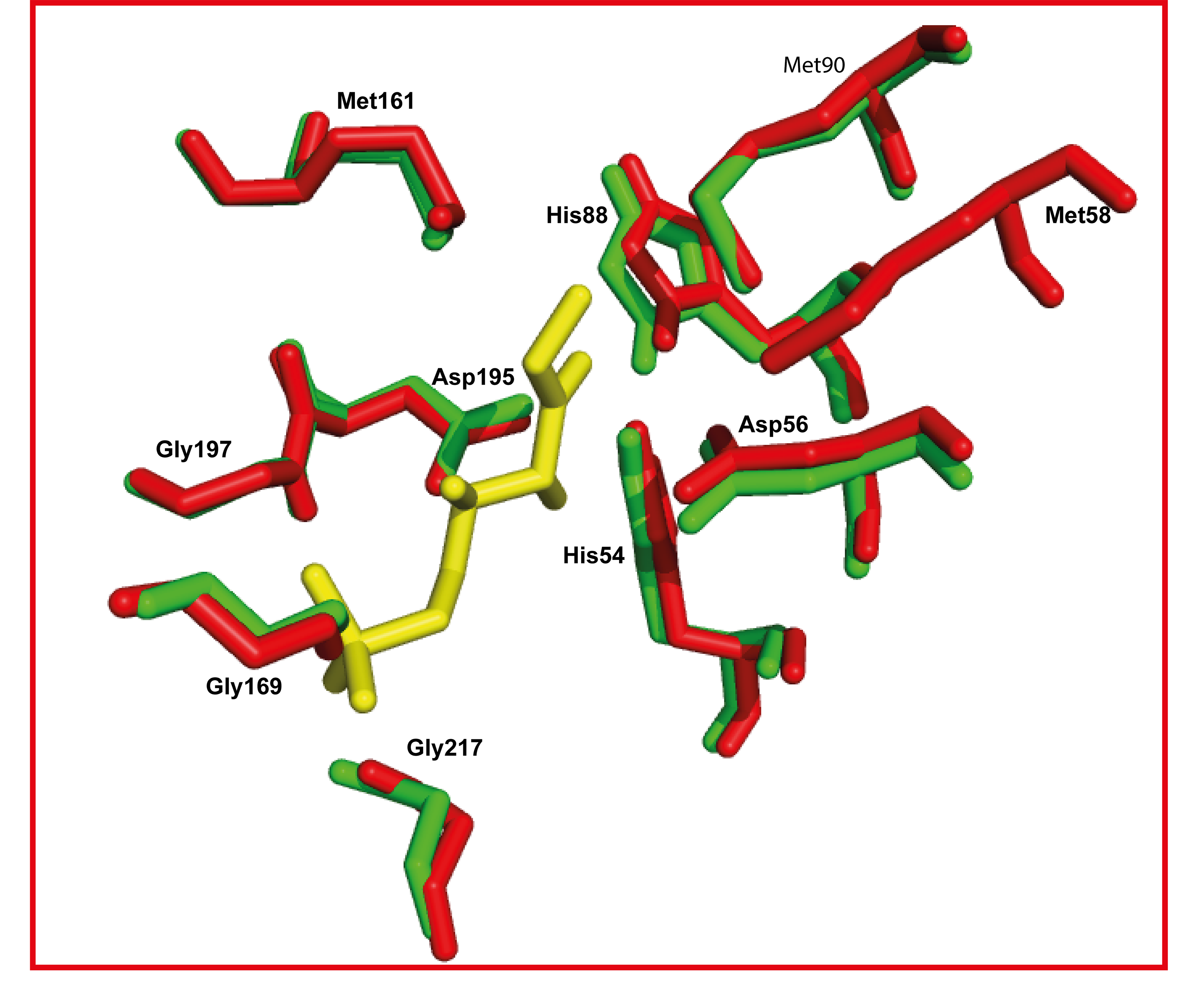

Supplement: S10 Fig — Superimposition of the active sites of TcRPE1 and TcRPE2 models. Important amino acid residues for catalysis and substrate docking are shown in red sticks for TcRPE1 and in green sticks for TcRPE2. Ribulose 5-phosphate is shown as yellow sticks. The S12 amino acid residue is not shown to attain clear visualization of the active site residues. (TIF) [file pone.0172405.s010.tif]

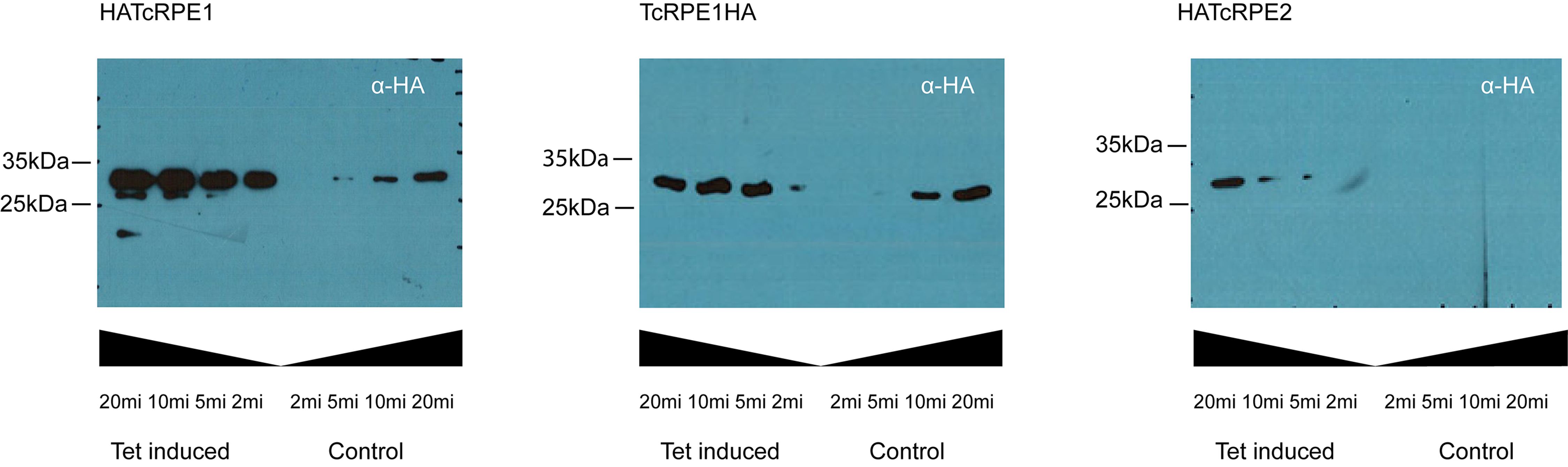

Supplement: S11 Fig — Western blot of cell free extracts from HATcRPE1, TcRPE1HA, and HATcRPE2 CL Brener [pLEW13] lines after 72 h induction with tetracycline, with rat anti-HA antibodies. Different amounts of induced and non induced (control) parasites -ranging from 2 to 20 millions- were loaded to attain clear visualization. (TIF) [file pone.0172405.s011.tif]

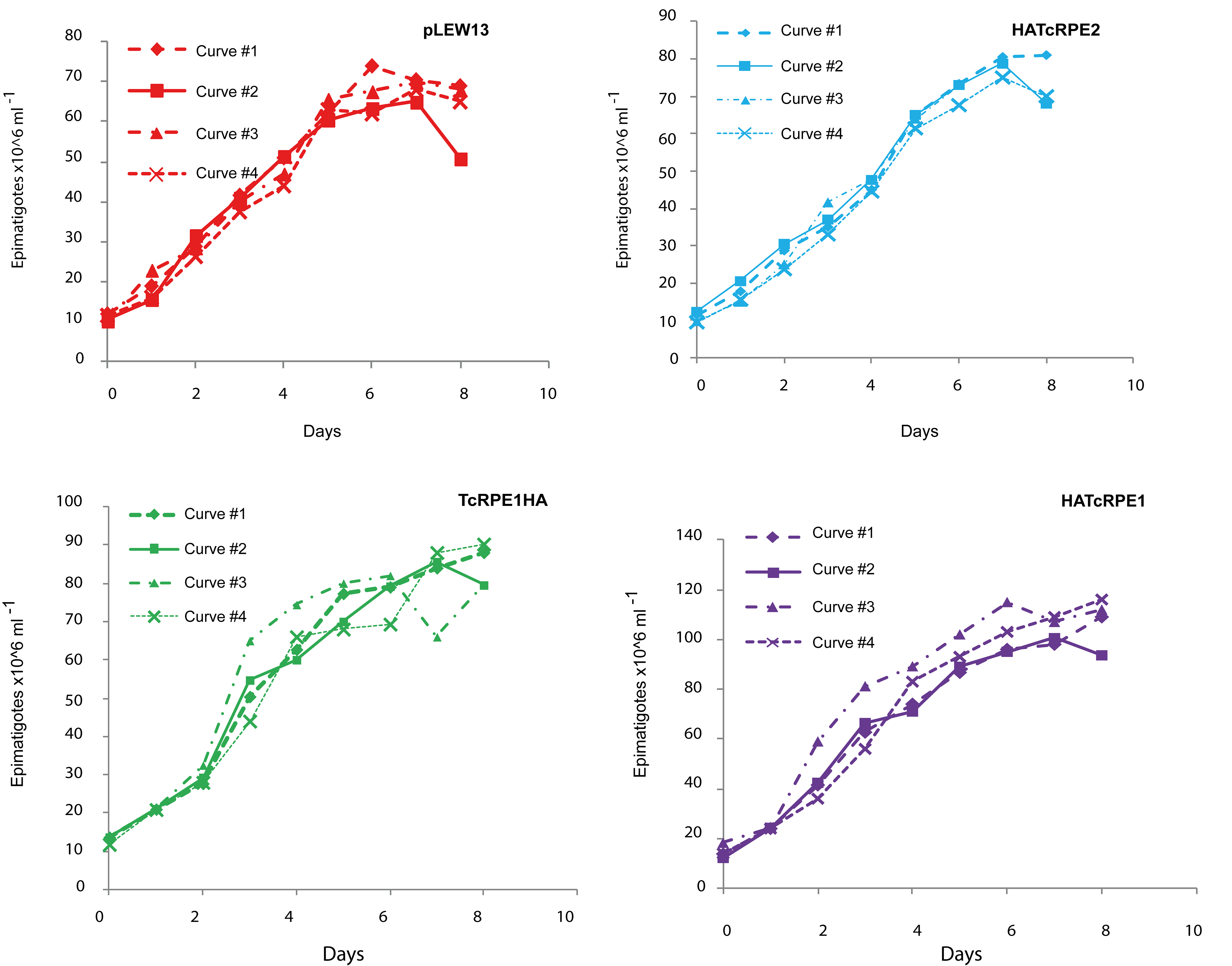

Supplement: S12 Fig — Parasites were grown in BHT medium and followed for 8 days until the stationary phase was reached. Four independent experiments performed for each transfected line are shown. (TIF) [file pone.0172405.s012.tif]

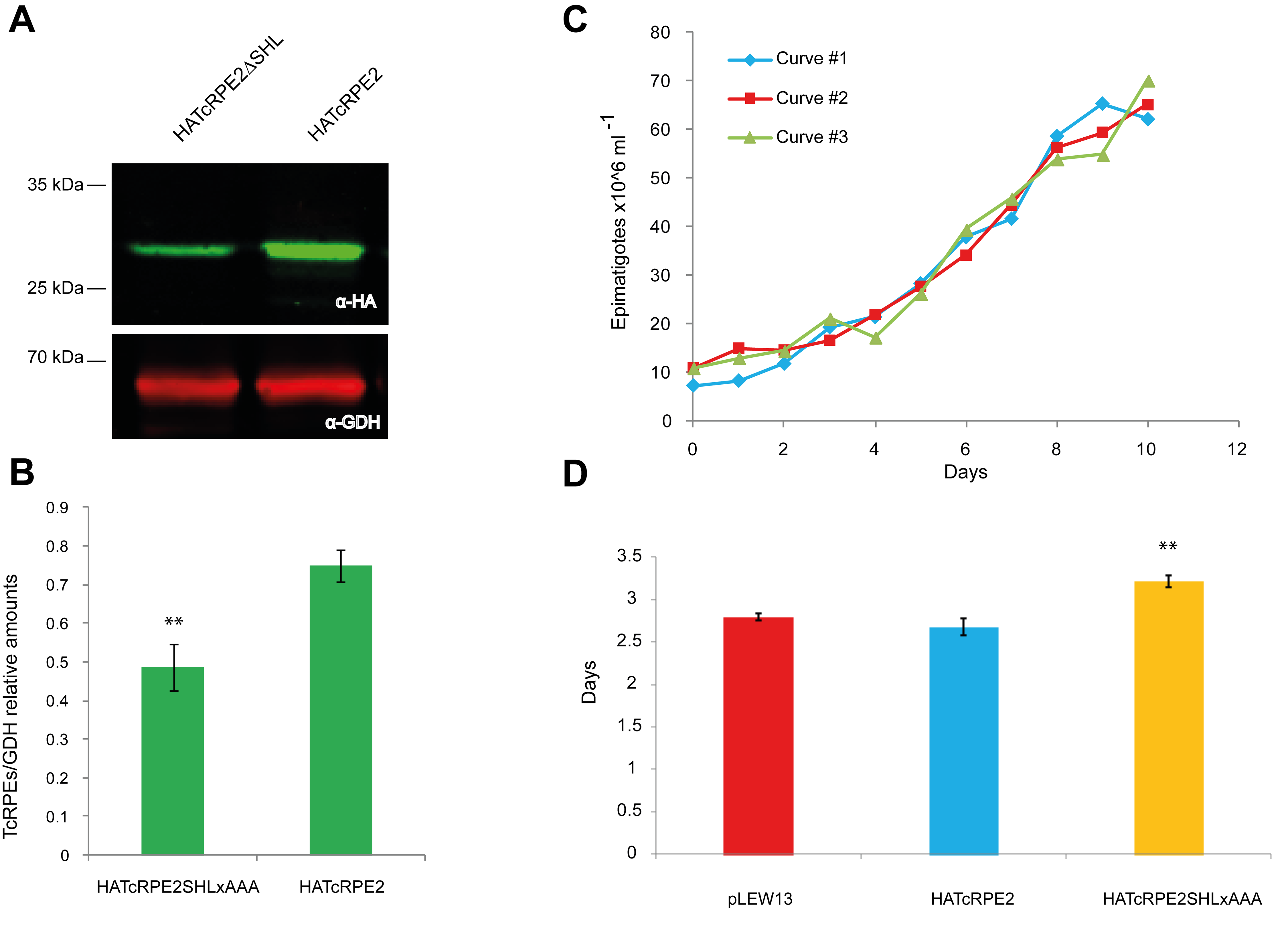

Supplement: S13 Fig — (A) Western blot of cell free extracts from HATcRPE2, HATcRPE2SHLxAAA lines after 72 h induction with tetracycline, with rat anti-HA antibodies and rabbit anti-GDH antibodies as loading control.(B) The intensity of the HATcRPE2 and HATcRPE2SHLxAAA bands was quantified from three independent experiments and normalized to α-GDH intensity. The bar graph represents the mean ± SEM of the relative intensity of the bands.(C) Gowth curves of HATcRPE2SHLxAAA strain under tetracycline induction. Parasites were grown in BHT medium and followed for 10 days until the stationary phase was reached. Three independent experiments were performed, named Curve #1–3. (D) Doubling times of the pLEW13, HATcRPE2 and HATcRPE2SHLxAAA lines were calculated from three independent experiments. The bar graph represents the mean ± SEM of the doubing time. (TIF) [file pone.0172405.s013.tif]
